# Functional expression of the entire adhesiome of *Salmonella enterica* serotype Typhimurium

**DOI:** 10.1038/s41598-017-10598-2

**Published:** 2017-09-04

**Authors:** Nicole Hansmeier, Katarzyna Miskiewicz, Laura Elpers, Viktoria Liss, Michael Hensel, Torsten Sterzenbach

**Affiliations:** Abteilung Mikrobiologie, Fachbereich Biologie/Chemie, Universität Osnabrück, Barbarastr. 11, 49076 Osnabrück, Germany

## Abstract

Adhesins are crucial virulence factors of pathogenic bacteria involved in colonization, transmission and pathogenesis. Many bacterial genomes contain the information for a surprisingly large number of diverse adhesive structures. One prominent example is the invasive and facultative intracellular pathogen *Salmonella enterica* with an adhesiome of up to 20 adhesins. Such large repertoire of adhesins contributes to colonization of a broad range of host species and may allow adaptation to various environments within the host, as well as in non-host environments. For *S*. *enterica*, only few members of the adhesiome are functionally expressed under laboratory conditions, and accordingly the structural and functional understanding of the majority of adhesins is sparse. We have devised a simple and versatile approach to functionally express all adhesins of *S*. *enterica* serotype Typhimurium, either within *Salmonella* or within heterologous hosts such as *Escherichia coli*. We demonstrate the surface expression of various so far cryptic adhesins and show ultrastructural features using atomic force microscopy and transmission electron microscopy. In summary, we report for the first time the expression of the entire adhesiome of *S*. *enterica* serotype Typhimurium.

## Introduction

Pathogenic bacteria possess a plethora of virulence factors to elegantly manipulate hosts and thereby induce the development of diseases. Indispensable for colonization and establishment of their pathogenic potential are adhesive structures, of which most pathogens display a complex arsenal ranging from single molecules up to highly elaborated macromolecular nanomachines^1, 2^. For example *S*. *enterica* encodes, depending on the serotype, for up to 13 fimbrial and at least 7 non-fimbrial adhesins^3, 4^, pathogenic *E*. *coli* for up to 14 fimbrial and at least 10 non-fimbrial adhesins^5, 6^, *Yersinia pestis* for at least 13 autotransported and 8 fimbrial adhesins^7^ and *Mycobacterium tuberculosis* for at least 20 known adhesins^8^.

The complexity of adhesin display not only adapts depending on the host environment but rather also determines the induction and outcome of different diseases. For example fimbriae are crucial for the development of bladder and kidney infections or enterocolitis caused by uropathogenic or enterotoxigenic *Escherichia coli* ^9–11^. In *Salmonella*, fimbriae are required for long-term colonization of mice^12^. In *Yersinia spp*. and enteropathogenic or enterohemorrhagic *E*. *coli* the inverse autotransporter adhesins invasin and intimin are indispensable for invasion, protein translocation or colonization of the host^7, 13^. Expression of adhesins is restricted to the appropriate time or location since their untimely expression can be detrimental for bacterial colonization and pathogenesis^14^. Furthermore, once expressed they can become one of the most abundant proteins in the cell^15^. Therefore, their expression is tightly regulated at various levels. This is one reason why most adhesins are not expressed in laboratory cultures. In *S*. Typhimurium for example, the only adhesins readily expressed under laboratory conditions are type 1 fimbriae, curli fimbriae and the type 1 secretion system-secreted adhesin SiiE^16–18^, while most of them are expressed in animal models^19^. Furthermore, some adhesins (i.e. Pef, Std, MisL) can be expressed in certain regulatory mutants^14, 15, 20–22^. As a consequence, generally little is known about their structure, binding partners, and roles in pathogenesis and bacterial lifestyle.

To overcome these obstacles, previous studies have used IPTG- or arabinose-inducible systems for the expression of adhesins in, for example, *Yersinia* spp. or pathogenic *E*. *coli* ^23–27^. In this study, we devised and optimized a tetracycline-controlled expression system enabling ‘on demand’ expression of adhesins. We demonstrate for the first time the expression of the adhesiome of *S*. Typhimurium. Furthermore, we characterized the synthesized adhesins by atomic force microscopy (AFM) and transmission electron microscopy (TEM), providing the description of structural appearance of divergent adhesins.

## Results

### Expression system for previously uncharacterized adhesins in *S*. Typhimurium

**Figure 1:**
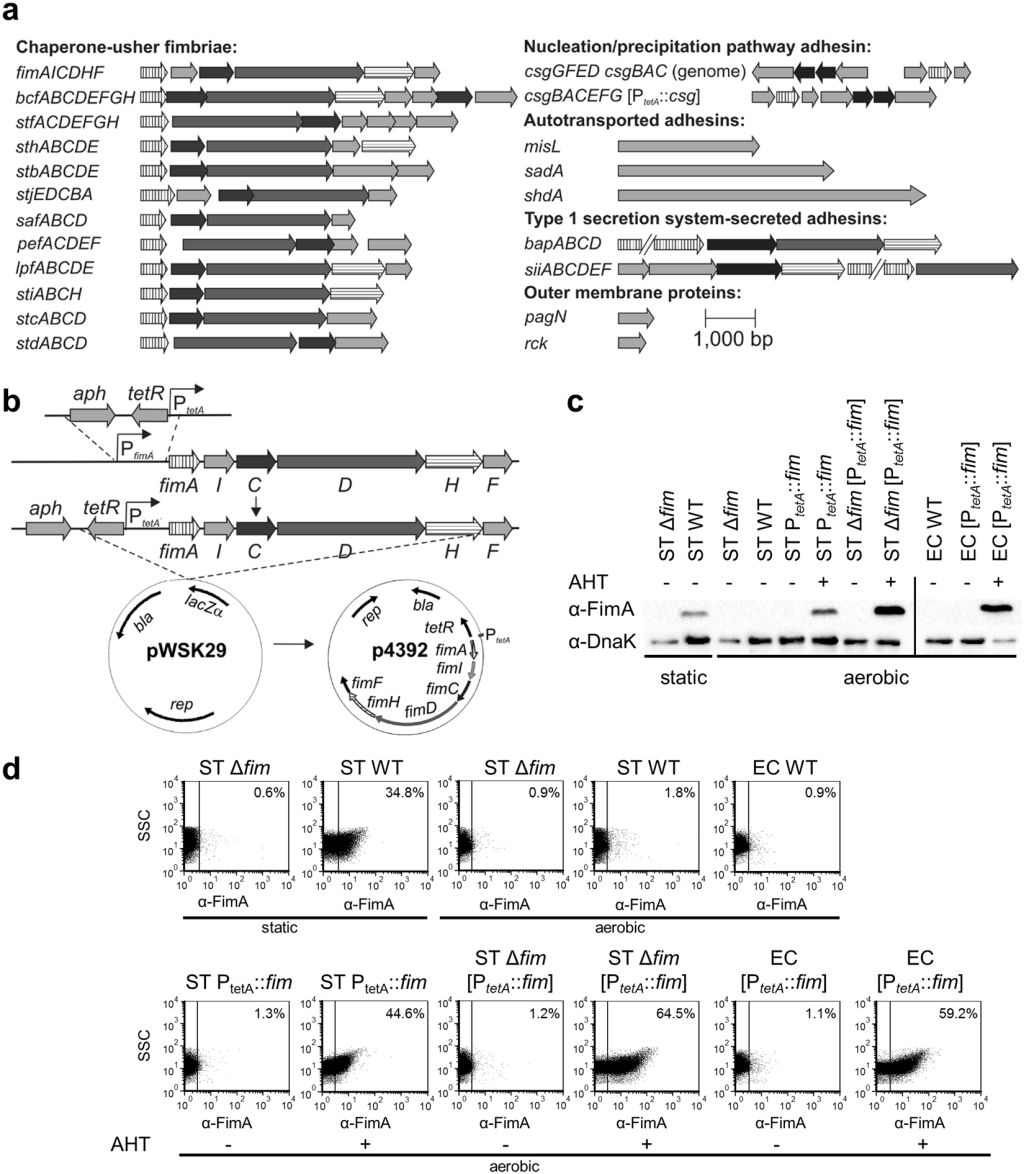
Overview of the adhesiome of *Salmonella enterica* serotype Typhimurium and the approach for controlled expression of the adhesiome. (**a**) Genetic organization of operons and genes predicted to encode for adhesins in *S*. Typhimurium. Vertically hatched arrows: main subunits (CU fimbriae and Nucleation/precipitation pathway) or substrates (T1SS); horizontally hatched arrows: predicted tip adhesins (CU fimbriae) or periplasmic adapter proteins (T1SS); dark grey arrows: usher proteins (CU fimbriae) or ATPases (T1SS); black arrows: chaperone (CU fimbriae or Nucleation/precipitation pathway) or outer membrane proteins (T1SS); light grey arrows: other components. Arrows depicting substrates of T1SS are truncated. For operons encoding for curli fimbriae (Nucleation/precipitation pathway) the upper and lower scheme depicts the organization in the genome of *S*. Typhimurium and the organization in the expression construct P_*tetA*::_ *csg*, respectively. (**b**) Scheme of the strategy for controlled expression of the adhesiome as exemplified for type 1 fimbriae. Promoters in front of operons or genes encoding for adhesins were exchanged with a tetracycline-inducible expression cassette. For plasmid-borne expression, the promoter cassette and operons or genes encoding for adhesins were inserted into pWSK29. (**c** and **d**) Controlled expression of type 1 fimbriae. Strains as indicated were grown either statically for 24 h or in a roller drum for 3.5 h in the presence ( + ) or absence of 100 ng/ml of the inducer AHT. (**c**) Expression of the main subunit of type 1 fimbriae FimA or DnaK was detected by Western blotting from lysates of the indicated strains. Full-length blots including molecular size markers are presented in Supplementary Fig. 9. (**d**) Surface expression of type 1 fimbriae in the indicated strains was assessed by flow cytometry targeting the main subunit FimA.

Based on genome data, *S*. Typhimurium encodes 12 chaperone-usher (CU) fimbriae^4, 28^, curli fimbriae, which are assembled by the nucleation/precipitation pathway^29^, two adhesins secreted by a type 1 secretion system (T1SS), i.e. BapA and SiiE^30, 31^, three autotransported adhesins, i.e. MisL, SadA and ShdA^32–34^ and two atypical adhesins, i.e. Rck and PagN^35, 36^ (overview in Fig. 1a). Since most of them are not expressed under laboratory conditions, we decided to express the operons or genes encoding for all known adhesins in *S*. Typhimurium under control of a tetracycline-inducible promoter element. The putative promoter sequences of candidate genes or operons were replaced on the chromosome by a gene cassette containing a selectable resistance marker, *tetR* encoding the Tet repressor, and the *tetA* promoter (P_*tetA*_) (Fig. 1b). For ectopic expression of adhesive structures, we transferred the respective genes or operons encoding for adhesins and the tetracycline-inducible promoter onto the low copy-number plasmid pWSK29. BapA as well as SiiE and their cognate T1SS could not be successfully transferred into pWSK29, probably due to their length (16 kb for *bapABCD* and 23 kb for *siiABCDEF*). Since the genes encoding for *shdA* and *lpfD*, the tip adhesin of Lpf fimbriae, are pseudogenes in *S*. Typhimurium NCTC 12023, we amplified *shdA* and the *lpf* operon from the genome of *S*. Typhimurium LT2. All constructs were then confirmed by sequencing. As a host for expression of adhesins, we used *S*. Typhimurium SR11^37^ and *E*. *coli* ORN172^38^, since these strains are commonly used for expression of adhesins.

### Characterization of the expression system

We first validated the expression system for type 1 fimbriae, since these are the best characterized CU fimbriae of *S*. Typhimurium and can be readily expressed in wild-type (WT) *Salmonella* under static growth conditions. After growing WT *S*. Typhimurium (ST) statically for 48 h, we detected expression of the main subunit of type 1 fimbriae FimA by Western blotting, while there was no expression in an *S*. Typhimurium *fimAICDHF* deletion strain, or in *E*. *coli* ORN172 (EC) (Fig. 1c). Also under aerobic cultivation, we could not detect expression of FimA in ST WT. After addition of anhydrotetracycline (AHT), we detected FimA in ST P_*tetA*_::*fim* harboring the chromosomal encoded tetracycline-inducible promoter upstream of the *fimAICDHF* operon, as well as in an ST Δ*fimAICDHF* strain or EC harboring plasmid p4392 for expression of *fimAICDHF*, while no signal was detected in non-induced controls. To optimize the tetracycline-inducible system, we then induced these strains with increasing concentrations of AHT, ranging from 1–300 ng/ml (Fig. S1a–c). We detected expression of FimA in Western blotting after induction with AHT starting at 3–10 ng/ml, while reaching its maximum with 30 ng/ml AHT.

Next, we confirmed surface expression of type 1 fimbriae by flow cytometry (Fig. S1d,e,f). Approximately 35% of ST WT were positive for expression of type 1 fimbriae after static growth when using a *fimAICDHF* deletion mutant for gating. In ST P_*tetA*_::*fim* and ST Δ*fimAICDHF* [P_*tetA*_::*fim*] or EC [P_*tetA*_::*fim*], approximately 44% to 65% of bacteria were positive for surface expression of type 1 fimbriae after AHT induction (Fig. 1d). Various concentrations of the inducer AHT were then again used to define conditions for optimal surface expression. Here, type 1 fimbriae were detectable after induction with 1–3 ng/ml AHT and the maximal expression was reached with 100 ng/ml AHT (Fig. S1d,e,f). Consequently, we used 100 ng/ml AHT for further experiments.

Then we visualized surface expression of type 1 fimbriae in ST P_*tetA*_::*fim* as well as in ST Δ*fimAICDHF* [P_*tetA*_::*fim*] or EC [P_*tetA*_::*fim*] by AFM (Fig. S2). When WT SR11 was grown statically, type 1 fimbriae were visible on the surface of the bacteria, while as expected no fimbriae were visible on SR11 Δ*fimAICDHF* or EC WT. Type 1 fimbriae were also clearly detected on the surface of induced bacteria in ST P_*tetA*_::*fim* or SR11 Δ*fimAICDHF* [P_*tetA*_::*fim*] and EC [P_*tetA*_::*fim*]. Next, we measured the diameter of the fimbriae. The fimbriae had in all cases a diameter of approximately 7 nm on average, which is in concordance with previously published data^39, 40^.

To test the functionality of type 1 fimbriae, we assayed binding to mannose as natural ligand^41^. When we expressed type 1 fimbriae in EC WT, we could detect increased adherence to mannose-BSA-coated surfaces compared to non-induced samples or EC WT (Fig. S3a). Since expression of type 1 fimbriae did not lead to increased adherence to surfaces coated with BSA alone, we verified that binding was specific to mannose.

These results clearly prove that the deployed expression system was suitable for the expression of functional fimbriae in both *Salmonella* as well as heterologous hosts.

### Expression of previously uncharacterized adhesins of *S.* Typhimurium

Next we employed the tetracycline-inducible expression system for the remaining adhesins of *S*. Typhimurium. Since *E*. *coli* ORN172 encodes for fewer pronounced surface structures allowing better visualization of the respective adhesins and was previously used for expression of chaperone-usher fimbriae in other studies^19, 20, 23, 42–44^, we decided to use this strain as a host to express the adhesiome of *S*. Typhimurium.

We first analyzed the expression of 17 adhesins by Western blot and flow cytometry. According to the previous defined parameters, we used 100 ng/ml AHT for induction of expression of adhesins. For most adhesins we detected a specific band at the size of the main fimbrial subunit (for CU-fimbriae), substrates (for T1SS), autotransporters, or outer membrane proteins after AHT induction of the respective adhesin in Western blots, while no signals were observed in ORN172 or in non-induced controls (Fig. S4). However, for Lpf, Saf, Bcf and Stb fimbriae, expression of the respective main fimbrial subunits was not detectable. Similar results were obtained when we analyzed surface expression of adhesins by flow cytometry. In most cases, we detected a population with increased fluorescence signals after AHT induction (7–90% positive cells) in comparison to ORN172 or non-induced controls (Fig. S5). This indicates that these adhesins were successfully expressed on the bacterial surface. In accordance with Western blotting, either no or very low expression was detected by flow cytometry for Lpf, Saf, Bcf and Stb fimbriae. It is possible that these fimbriae could not be successfully expressed or that the antisera were not suitable for detection of the respective native fimbriae. We were also not able to detect a signal for PagN or Rck in flow cytometry, while these proteins were readily detectable by Western blotting. For these later candidates, we assume either that the corresponding antibodies are unable to detect the native adhesins or that their structures might be too small to protrude the LPS layer so that the required epitopes were not sufficiently accessible for antibody binding.

### Visualization of non-fimbrial adhesins of *S*. Typhimurium

We then visualized non-fimbrial adhesins of *S*. Typhimurium using TEM and AFM, respectively. This approach provides the advantages of both techniques: visualization of adhesins on living bacteria under ambient conditions (AFM), determination of adhesin properties such as diameter and length (AFM), as well as ultrastructural resolution (TEM) with potential immuno-labeling.

**Figure 2:**
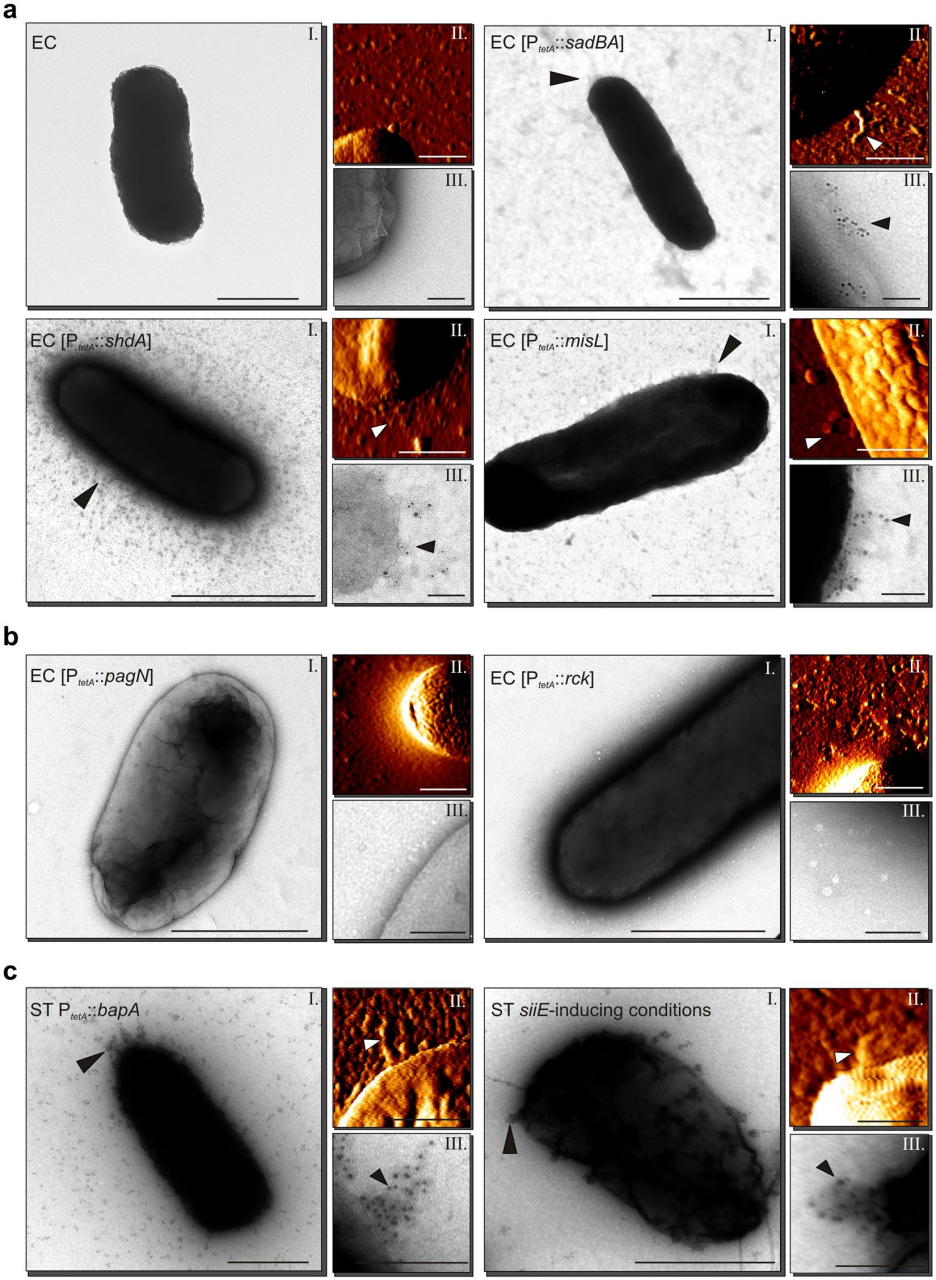
Visualization of non-fimbrial adhesins of *S*. Typhimurium by AFM and TEM in *E*. *coli* ORN172 (**a** and **b**) or *S*. Typhimurium (**c**). Each adhesin is shown in panels with a TEM image (I), an AFM deflection image (II) and a TEM image of immuno-gold labeled cells (**a** and **c**) or non-labeled cells (**b**) (III). Arrow heads point to the respective adhesive structure. Scale bars indicate 0.5 µm (I and II) or 0.1 µm (III).

After expression of autotransporters MisL, SadA and ShdA in *E*. *coli* WT, we detected very short appendages on the surface of the bacteria by TEM and AFM (Fig. 2a). To verify that these structures represent autotransporter adhesins, bacteria were labelled by adhesin-specific antisera and immuno-gold (Fig. 2aIII). We observed that the previous detected structures were henceforth labeled in bacteria expressing SadA, ShdA or MisL with specific sera. Furthermore, functional MisL and ShdA are known to bind specifically to fibronectin^32, 45^. Therefore, we decided to test their functionality for fibronectin-binding. While there was no difference in adherence to BSA-coated plates, we could detect increased adherence to fibronectin-coated plates in EC expressing *shdA* or *misL* compared to EC WT (Fig. S1b). Thus, we concluded that ShdA and MisL were functionally expressed on the surface of EC.

Short filamentous appendages were visualized on the surface of bacteria upon expression of PagN or Rck with microscopy approaches (Fig. 2b), which were absent in controls. These structures may be PagN or Rck, two OMPs that were suggested to be involved in SPI1-T3SS-independent invasion of epithelial cells^36, 46^. However, since they could not be verified by flow cytometry or immunogold-labeling, further studies are required to identify the nature of the observed structures.

BapA and its cognate T1SS were expressed in *S*. Typhimurium NCTC 12023 (Fig. 2c). We detected small filaments on the surface of the bacteria, which were labeled by immuno-gold. BapA is involved in biofilm formation^31, 47^ and in assays for biofilm formation, we detected significantly enhanced biofilm formation in bacteria expressing *bapA* compared to non-induced bacteria or ST WT (Fig. S1c), confirming functionality of BapA and its cognate T1SS. The *sii* operon encoding giant adhesin SiiE and its cognate T1SS is expressed during late exponential growth in rich media, thus expression under control of the native promoter was deployed. Similar to BapA we observed small filaments on the bacterial surface which were decorated with immuno-gold (Fig. 2c). Overall, these results demonstrate the expression and functionality of most of the analyzed non-fimbrial *S*. Typhimurium adhesins.

### Visualization of fimbrial adhesins of *S*. Typhimurium

Fimbriae are known to exhibit diverse morphologies. Some form rather typical rod-like structures as the paradigmatic type 1 or Pap fimbriae of uropathogenic *E*. *coli*, while others form rather atypical bulky or capsular structures composed of very thin fibrillae as for example Afa, CS3 or CS6 of pathogenic *E*. *coli* or the F1 capsule of *Y*. *pestis* ^48–54^.

**Figure 3:**
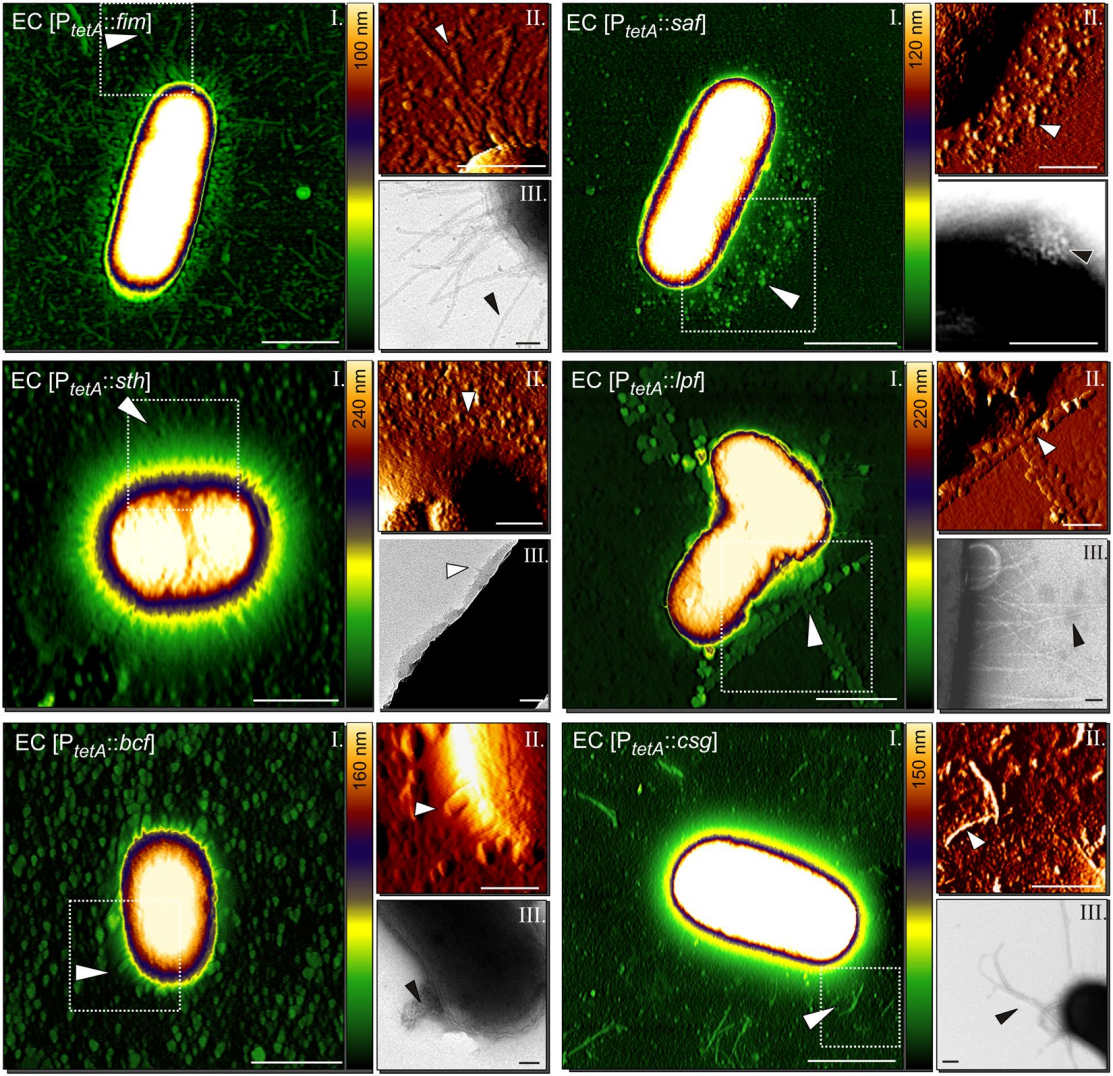
Visualization of thick fimbrial adhesins of *S*. Typhimurium by AFM and TEM. Each adhesin is shown in panels with an AFM height image (I), an AFM deflection image (II) and a TEM image (III). Arrow heads point to the respective adhesive structure. Hatched boxes in (I) indicate the area depicted in AFM deflection images (II). Color bar indicates the Z-range. Scale bars indicate 1 µm (I), 0.5 µm (II), or 0.1 µm (III). Example height plots from each fimbrial adhesin are shown in Suppl. Fig. S7.

Despite the presence of a large number of operons encoding for fimbrial adhesins in *Salmonella*, only few of these have been visualized and functionally characterized before. Best characterized are type 1 fimbriae and curli fimbriae. Curli are fibrous, loosely cell surface-attached structures, assembled by the nucleation/precipitation pathway and commonly expressed in diverse *S*. *enterica* serovars.^55, 56^. Curli were previously visualized in *S*. Enteriditis and *S*. Typhimurium by TEM^55, 56^ and shown as very thin, flexible and highly intertwined self-aggregative filaments. We observed similar structures both by AFM and TEM (Fig. 3). Furthermore, we detected increased biofilm formation by bacteria expressing curli fimbria compared to non-induced samples or EC WT (Fig. S3d). These results confirmed functional expression of curli fimbriae.

**Figure 4:**
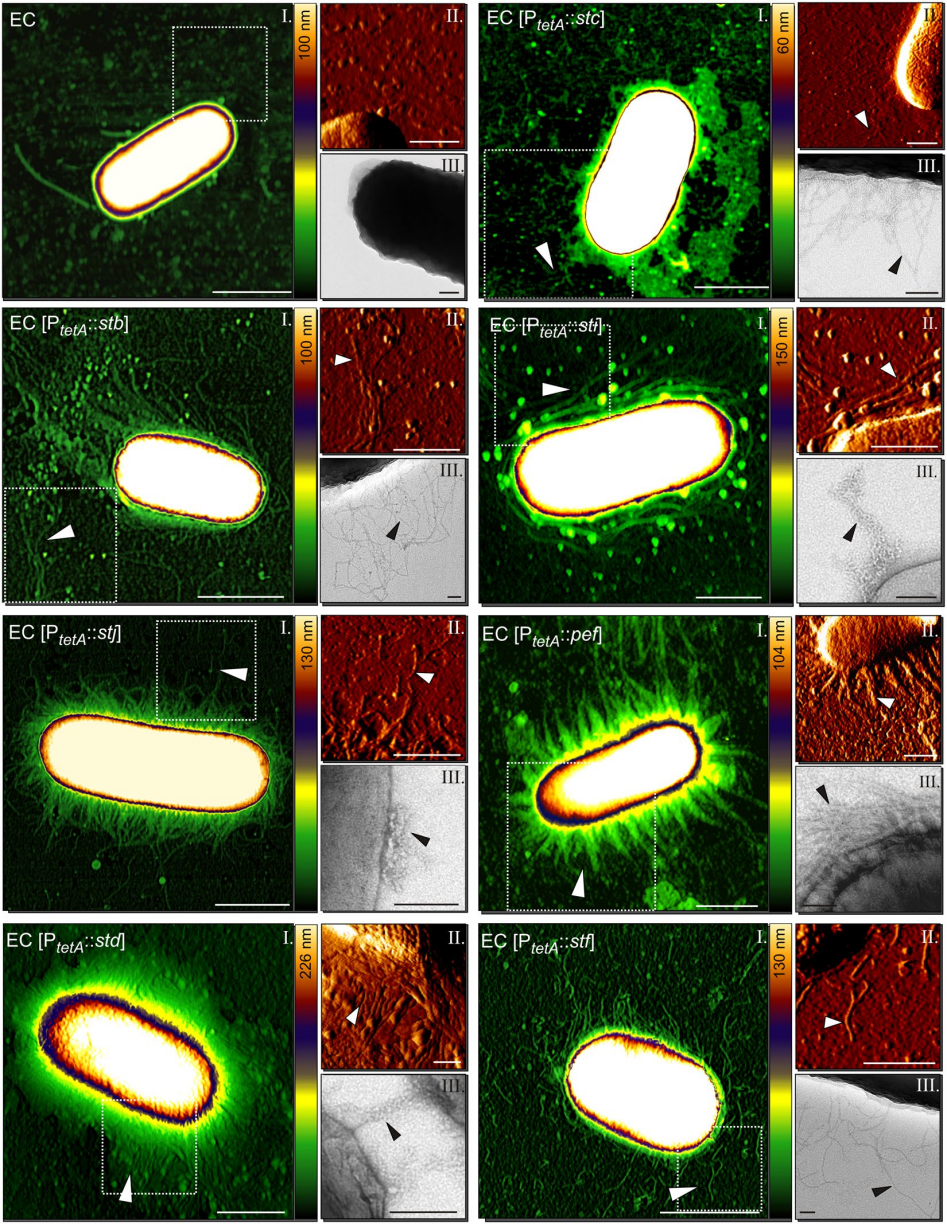
Visualization of thin fimbrial adhesins of *S*. Typhimurium by AFM and TEM. Each adhesin is shown in panels with an AFM height image (I), an AFM deflection image (II) and a TEM image (III). Arrow heads point to the respective adhesive structure. Hatched boxes in (I) indicate the area depicted in AFM deflection images (II). Color bar indicates the Z-range. Scale bars indicate 1 µm (I), 0.5 µm (II), or 0.1 µm (III). Example height plots from each fimbrial adhesin are shown in Suppl. Fig. S7.

Using our combinatory approach, we visualized the remaining 12 *Salmonella* fimbrial adhesins and found that the appearance of the fimbrial structures varied widely (Figs 3 and 4). We observed typical fimbrial structures characterized by long straight (Fim, Std) or slightly curved (Stb, Stc, Stf, Sti, Stj, Pef) appendages. Similar structures were reported in diverse bacteria e.g. *K*. *pneumoniae* type 3 fimbriae^57^ and in *S*. Typhimurium Pef^58, 59^, Std^60^ and type I fimbria^39, 40^. We also identified atypical fimbria (Lpf, Saf, Bcf, Sth). Comparable structures for Lpf and Saf fimbriae of S. Typhimurium have been previously reported^61, 62^. Similar morphologies as for Lpf, Bcf and Saf fimbriae were also observed in other bacteria as for instance for long polar, CS3 or Afa fimbriae of pathogenic *E*. *coli* ^50, 54, 63^. Furthermore, the localization of the fimbria was similarly variable. Some fimbriae appeared peritrichous such as type I fimbriae, others formed bundles such as Pef fimbriae, further fimbriae appeared to be limited to one side of the bacterium such as Saf fimbriae.

To verify that the observed structures were indeed formed by overexpressed fimbrial proteins, we performed immuno-EM with nano-gold particles followed by silver enhancement. With the exception of Sth and Lpf fimbriae we detected specific staining of the respective fimbrial structures (Fig. S6), confirming the identity of the fimbriae. Since expression of Sth and Lpf fimbriae was neither detected by Western Blotting nor by flow cytometry, we assume that the lack of specific staining is due to low quality of the primary antibodies.

**Figure 5:**
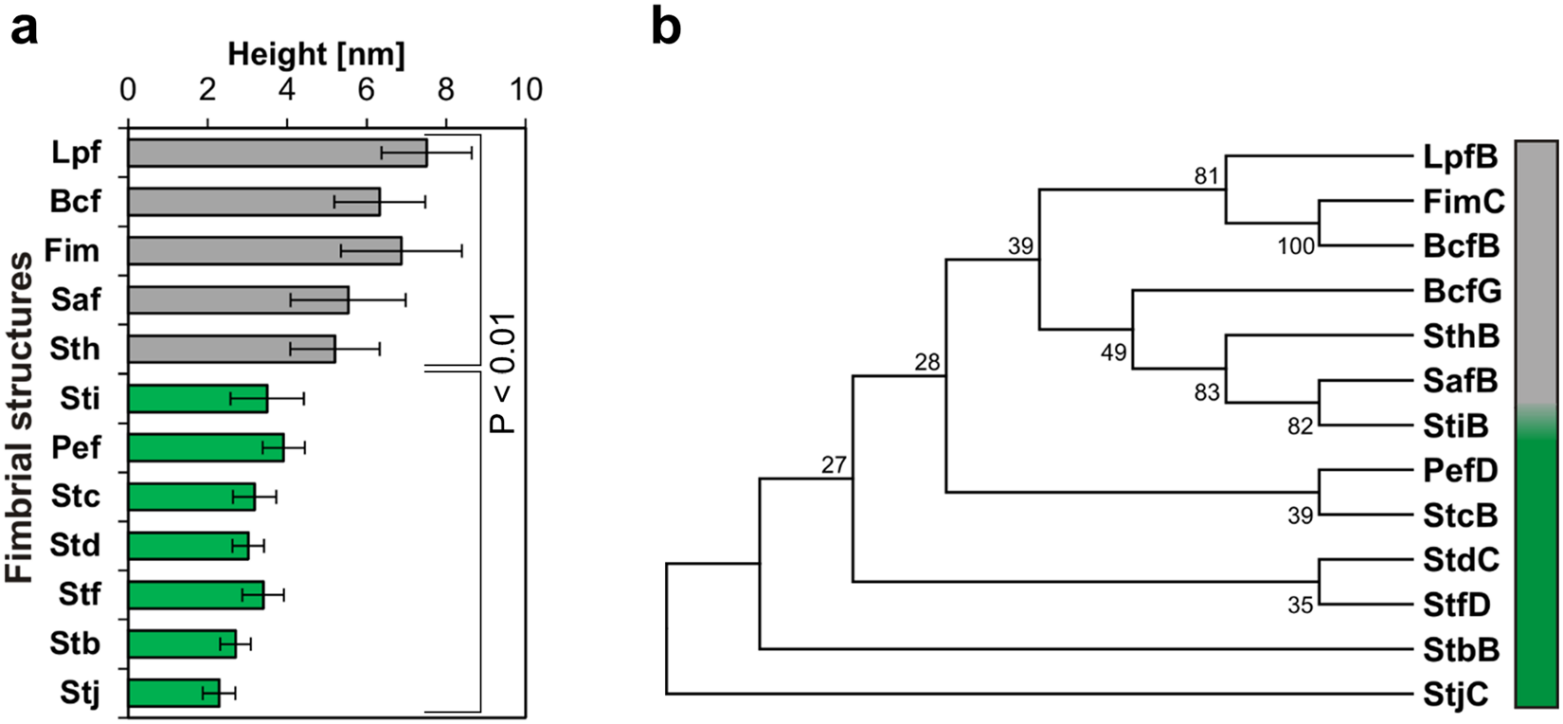
Characterization of (**a**) thin and (**b**) thick fimbrial adhesins of *S*. Typhimurium. (**a**) The diameter of 20 randomly selected fimbrial structures from three biological replicates each were measured from AFM height images. The graphs show the average height and the standard deviation of the respective fimbrial structure. Grey bars indicate thick (C_Thick_) and green bars thin (C_Thin_) fimbriae. Statistical analyses using t-test indicate that these two groups (C_Thin;_ C_Thick_) are statistically different. (**b**) Bootstrap consensus tree of fimbrial chaperone proteins inferred from 500 replicates generated by MEGA7 using the Neighbor-Joining method. The percentage of replicate trees in which the associated taxa clustered together in the bootstrap test are shown next to the branches.

Next, we determined the fimbrial diameters by AFM and TEM (Fig. 5a, S7, S8). The positions of measurements were carefully chosen to ensure that individual fimbria rather than bundles were measured. We observed that the twelve analyzed fimbria can be divided into two distinct groups. Fimbriae of the first group containing Bcf, Lpf, Sth, Fim and Saf exhibited diameters ranging from 5.2 ± 1.1 nm to 7.5 ± 1.1 nm, whereas fimbriae of the second group (Stb, Stc, Std, Stf, Sti, Stj and Pef fimbriae) showed slightly thinner diameters ranging between 2.3 and 3.5 nm as determined by AFM (Fig. 5a) and TEM (Fig. S8). Diameters of Fim^39, 40^, Lpf^61^ and Pef fimbriae ^58, 59^ were previously determined and are in accordance with our measurements.

We then generated a consensus tree using the amino acid sequences of the respective chaperone proteins encoded within fimbrial operons (Fig. 5b). We could clearly separate a distinctive branch consisting of chaperones LpfB, FimC, BcfB, BcfG, SthB, SafB and StiB. Interestingly, with the exception of Sti fimbriae, chaperone proteins of this branch belonged to the group with the thick diameters. In contrast, chaperone proteins of the remaining fimbrial structures clustered to different branches. All these adhesins belonged to the group with the thinner fimbrial diameters.

In summary, we expressed and visualized for the first time all fimbrial structures of *S*. Typhimurium. Furthermore, we detected a clear morphological distinction into thin and thick fimbriae, which might be associated with their primary sequence and their phylogenetic related chaperones.

## Discussion

Adhesion and biofilm formation are crucial factors for transmission, colonization and pathogenesis of bacteria. In line with this notion, many bacteria including S. *enterica*, encode for a large armory of different adhesins. Expression of these adhesins has to be limited to the appropriate time and environment^60^ and is tightly regulated and complex^15, 64, 65^. Therefore it is difficult to express adhesins under laboratory conditions. In this study we established an inducible expression system, which allowed us to express the whole set of adhesion factors of *S*. Typhimurium. Various expression systems that deployed arabinose- or IPTG-inducible promoters were used before for experimentally controlled expression of fimbriae^23–26^. However, arabinose-inducible expression systems use carbon metabolites for system induction, which might interfere with the cellular metabolic status and thereby the expression of fimbriae. Furthermore, full repression of arabinose-inducible expression systems is only reached in the presence of glucose. Therefore, we reasoned that a tetracycline-inducible expression system is superior, since expression levels are well adjustable and anhydrotetracycline is not metabolized or toxic for bacteria.

With this system at hand, we are able to characterize and visualize the entire adhesiome of *S*. Typhimurium. We choose a combination of AFM and TEM to display the adhesins, since both techniques exhibit complementary advantages. Previous studies established and optimized the application of AFM for the study of biological samples^66–70^. Furthermore, AFM is widely used in biological setups to determine interaction forces for instance between receptor and ligands^71–76^. However, in contrast to TEM, so far only few studies have utilized AFM before for visualization of fimbriae as for instance BapA and curli fimbriae^47, 77–79^.

Though, AFM has certain advantages compared to TEM. For instance, AFM allows the visualization of surface structures of live bacteria under native conditions without major preparatory steps such as fixation and centrifugation, thereby preventing preparation-borne artefacts. By this means, appearance, length and diameter of fimbriae and other surface structures can be determined without affecting the integrity of surface structures by preparatory steps. On the other hand, TEM visualization is performed under high vacuum using a high energy electron beam; and preservation (fixation) procedure can circumvent disintegration of fragile structures during imaging and allows for more detailed ultrastructural analysis. However, preparatory steps may affect structural appearance. Thus we found our study will benefit from the use of both complementary techniques.

Visualization of CU fimbriae showed that their appearance varied widely between different fimbriae in both length and diameter. Fimbriae were previously classified into different clades according to phylogenetic relationship of the primary sequence of their respective usher protein^4, 28^. Depending on the number of subunits per helical turn fimbriae can be further classified into thick rigid and thin flexible fimbriae. In our study we clustered fimbriae based on their respective chaperone proteins. We could detect a clade (C_Thick_) consisting of chaperones of Lpf, Fim, Bcf, Sth, Saf and Sti fimbriae which almost exclusively formed fimbriae of larger diameter. Chaperones belonging to the remaining fimbriae clustered into distinctive clades (C_Thin_) and exclusively formed thin, flexible fimbriae. Interestingly, with the exception of Saf fimbriae also all fimbriae belonging to the C_Thick_ clade belonged to the γ_1_ fimbrial clade on the basis of usher proteins while none of the C_Thin_ fimbriae belonged to the γ_1_ fimbrial clade. This might imply a correlation between the relationship of chaperone and/or usher proteins and the morphology of fimbrial structures.

It is important to note that genes or operons encoding adhesins are often hotspots for the development of pseudogenes if the adhesin function is no longer required. Host-restricted serotypes of *S*. *enterica* often acquire a large number of pseudogenes in operons encoding for CU fimbriae, probably due to specialization to specific hosts^80, 81^. For example, the genome of *S*. Typhi harbors 12 operons predicted to encode for CU fimbriae, of which 7–8, depending on the isolate, were predicted to contain pseudogenes^80^. Also for *S*. Typhimurium it was previously reported that genes encoding for the tip adhesin of Lpf fimbriae and for the autotransporter ShdA contain mutations leading to pseudogenes in *S*. Typhimurium ATCC14028^82^. We confirmed these mutations by sequencing. However, the use of strain *S*. Typhimurium LT2, which did not contain these mutations, allowed expression of functional Lpf fimbriae and ShdA. Hence, we could show that after experimental induction of any of the 12 operons of *S*. Typhimurium predicted to encode for CU fimbriae, fimbrial structures could be visualized, suggesting the functionality of all fimbriae.

In summary, we generated a set of strains that allows us, to our knowledge for the first time, to express the complete set of known protein adhesins in a pathogenic bacterium. Since different serotypes of *S*. *enterica* differ widely in their composition of CU fimbriae as well as of other adhesins^4, 80^, this system can also be easily transferred to express adhesins specific to other serotypes than *S*. Typhimurium. Most importantly, this system allows to express adhesins of all kind of pathogenic or non-pathogenic bacteria to gain a better understanding of the role of adhesins in pathogenesis and lifestyle both for *Salmonella* and other pathogens.

## Materials and Methods

### Bacterial strains and growth conditions

Bacterial strains used in this study are listed in Table S1. Unless otherwise stated, bacteria were routinely grown aerobically in lysogeny broth (LB) (10 g Bacto tryptone, 5 g Bacto yeast extract, 10 g NaCl per liter) or on LB agar plates (LB containing 1.5% Difco agar). If necessary, carbenicillin or kanamycin were added at 50 mg/l. For induction of adhesin expression, overnight cultures of respective strains were diluted 1:30 into fresh medium containing the indicated concentration of anhydrotetracycline and incubated for 3.5 h in a roller drum (New Brunswick), unless otherwise noted.

### Construction of strains and plasmids

Template vector p3773 was generated by PCR amplification of *tetR* P_*tetA*_ from pWRG99 using TetR-PtetA-For-SacI and TetR-PtetA-Rev-XhoI. The resulting fragment was digested by XhoI and SacI and subcloned in XhoI/SacI digested plasmid p2795. For introduction of a tetracycline-inducible promoter element upstream of the coding region for Bcf, Fim, Lpf, Pef, Saf, Stb, Stc, Std, Stf, Sti and Stj fimbriae as well as the Bap T1SS, an expression cassette was amplified from p3773 using oligonucleotides as listed in Table S4 and introduced into the genome of *S*. Typhimurium NCTC 12023 by λ Red mutagenesis harboring pWRG730 for the expression of Redαβγ. The coding regions for the respective fimbriae including the *aph tetR* P_*tetA*_ cassette and the vector pWSK29 were amplified with oligonucleotides as listed in Table S4 and purified by PCR purification (Qiagen). The reverse-amplified PCR product from vector pWSK29 and the respective PCR products encompassing the coding sequence for fimbrial adhesins were then assembled by Gibson assembly according to manufacturer’s protocols (NEB).

The genes encoding for MisL, SadA, Rck, PagN, CsgBAC, CsgEFG and the operon encoding for the *bapABCD* operon were amplified from *S*. Typhimurium NCTC12023. *shdA* and the *lpf* operone were amplified from *S*. Typhimurium LT2 with oligonucleotides listed in Table S4. The vector p4392 including the *aph tetR* P_*tetA*_ but excluding the *fimAICDHF* operon was then reverse amplified using oligonucleotides listed in Table S4. After PCR purification, reverse-amplified PCR product from p4392 and the respective PCR products containing the coding regions for the respective adhesins were assembled by Gibson assembly. To generate the construct for expression of curli fimbriae, both PCR products encompassing the operons *csgBAC* and csgEFG were assembled with the reverse-amplified PCR product from p4392 by Gibson assembly in a single step to generate one hybrid operon under control of P_*tetA*_.

### Western Blotting

Cultures were resuspended to 1.5 × 10^10^ bacteria/ml in SDS-PAGE loading buffer containing 0.1% glycine/HCl, pH 2.2 and boiled for 5 min. Afterwards, samples were neutralized by adding 1 M Tris-HCl, pH 7.1 to a final concentration of 100 mM. 10 µl of samples were loaded on an 8–14% SDS-PAGE gel (depending on the size of the target protein) and after electrophoreses samples were blotted onto a 0.22 µm nitrocellulose membrane using a semi-dry electrophoretic transfer unit (BioRad). Blots were incubated with the respective specific antiserum^19, 31–33, 35, 36, 83^ (Table S3) diluted 1:500 and detected with a goat anti-rabbit IgG antibody conjugated to horseradish peroxidase and an ECL detection kit (Pierce). Blots were visualized with a Chemidoc imaging system (BioRad).

### Flow cytometry

For analyses of surface expression of adhesins by flow cytometry, 6 × 10^8^ bacteria were washed once in PBS and then fixed for 20 min in 3% paraformaldehyde. Afterwards, bacteria were stained with the respective specific antiserum diluted 1:250 and a goat anti rabbit IgG antibody coupled to Alexa-Fluor488. Bacteria were measured with a FACSCalibur (Becton Dickinson) and analyzed using FCS Express (De Novo Software). A mutant strain devoid of the respective adhesin was always used as a negative control for gating.

### Atomic force microscopy (AFM)

For AFM analysis, 20 μl of three independent bacterial cultivations were spotted onto glass coverslips (Hartenstein). After 1 h, samples were rinsed with ultrapure water to remove media residues before mounted onto microscopy slides for immediate AFM imaging.

AFM measurements were conducted under ambient conditions using the NanoWizard II AFM system (JPK Instruments AG, Berlin, Germany) by driving the AFM in soft contact mode using silicon nitride AFM probes with a nominal force constant of 0.06 N/m (SiNi, Budget Sensors, Wetzlar, Germany). Scan rates were set to 1 Hz and images were acquired with a resolution of 512 × 512 pixels. For each sample, topographic overview images were taken before zoom-ins on cells or appendages were performed. Representative height and deflection images are displayed in false-color. All height images were XY tilt corrected, polynomial- fitted and unsharpened mask filtered to remove noise using JPK data processing software (JPK Instruments AG). Height dimensions of fimbriae were determined after XY tilt correction from raw images and presented as mean values (n = 20). The positions for the analysis were carefully chosen to ensure that individual fimbria rather than bundles were measured. The values were derived from Z-dimensions since X-and Y-measurements are affected by the tip geometry.

### Transmission electron microscopy

In order to collect sufficient number of bacteria for TEM analysis bacterial cultures after induction of expression and without (controls) were concentrated by centrifugation at 1,000 x g. To circumvent potential loss of proteins from the bacterial surface or defragmentation during centrifugation, first bacteria where fixed for 30 min with 2.5% glutaraldehyde in LB medium. Bacterial pellets were washed twice in MilliQ water to remove traces of salts present in LB medium and gently re-suspended in MilliQ water. 4 µl of bacterial suspension was dropped on a formvar/carbon coated TEM grids that were glow discharged using the plasma cleaner (Diener electronic) shortly before preparation. Drops of suspension were left for 1 min to allow absorption of bacteria and blotted with a filter paper. Grids were negatively stained with 0.5% uranyl acetate, or 0.5% phosphotungstic acid with pH adjusted to 7.4, or left without staining, blotted with filter paper and finally air dried. TEM analysis was performed using a Zeiss 902 system operating at 50 kV for samples without negative staining. High resolution analysis and photographs of adhesins were performed at the negatively stained samples using the Libra 120 (Zeiss) operating at 120 kV and equipped with the omega energy filter and 2,000 × 2,000 pixel digital camera (Tröndle). Some of micrographs were adjust for brightness and contrast using ImageJ or Photoshop software when necessary. Structures at bacterial surface after induced adhesin expression and controls were compared for both negative stains.

Measurements of fimbrial diameters were performed on micrographs from TEM at 120 kV using ImageJ for both negative stains separately. No significant differences were detected for both staining methods. Only measurements of clearly singular fimbrial structures were included in analysis. Selection of structures for measurements was based on contrast allowing to discriminate edges of structures. Each fimbrial structure was measured in at least three locations along a length.

For immuno-EM, 4 µl of bacterial suspensions after induction of expression and controls without expression were collected on grids as described above, directly from cultures or after centrifugation and re-suspension in 50 m M Tris-HCl, pH 7.4 buffer (TBS). Next grids were washed in (TBS), blocked in 2% normal goat serum (NGS) in TBS for 30 min and incubated for 1 h with primary antibodies against various adhesins as described for flow cytometry. All primary antibodies were used at dilutions of 1:1,000 in TBS supplied with 0.1% BSA-c (Aurion), followed by second antibody coupled with nano-gold (Aurion) at a dilution of 1:200. Nano-gold was further enhanced for TEM analysis with the silver enhancement kit (Aurion) as described in the manufacturer protocol. Samples were further processed and TEM data were acquired as described above.

### Mannose and fibronectin binding assay

For mannose, 96-well flat bottom microtiter plates were coated with 20 µg/ml mannose-BSA or BSA only in 50 mM bicarbonate buffer pH 9.5 at 37 °C for 2 h. For fibronectin, the microtiter plates were coated with 50 µl/well fibronectin or BSA in 100 mM Tris-HCl, pH 8 for 16 h. The wells were then blocked with 10 mg/ml BSA for 1 h. 100 µl of bacterial cultures adjusted to an OD_600_ of 1 were added to the wells and incubated at 37 °C for 1 h. Unbound bacteria were removed by washing with PBS and bound bacteria were then visualized with wide-field microscope (Zeiss) using a 40 x objective. The number of bacteria per visual field were enumerated using ImageJ.

### Biofilm assay

Bacterial strains as indicated were diluted 1:100 from overnight cultures into fresh LB medium lacking NaCl and 200 µl of culture were inoculated in 96-well flat bottom microtiter plates for 48 h at 30 °C. Planktonic bacteria were then removed by three washes with PBS. Afterwards, adherent biofilm forming bacteria were stained with 0.1% crystal violet. The wells were then washed three times with H_2_O and crystal violet was solubilized with 30% acetic acid. The amount of crystal violet was afterwards quantified by measuring the absorbance at OD_595_.

### Bioinformatics analyses

A bootstrap consensus tree of fimbrial chaperones inferred from 500 replicates was constructed using Mega7^84^ using the Neighbor-Joining method^85^. Branches corresponding to partitions reproduced in less than 50% bootstrap replicates were collapsed. The percentage of replicate trees in which the associated taxa clustered together in the bootstrap test are shown next to the branches. The evolutionary distances were computed using the Poisson correction method^86^ and are in the units of the number of amino acid substitutions per site. All positions containing gaps and missing data were eliminated. There were 193 positions in total in the final dataset.

Statistical analysis was performed by a two-sample Student’s t-test with unequal variance.

## Electronic supplementary material

Supplementary Figures and Tables
