# Supplementary material for: Functional expression of the entire adhesiome of Salmonella enterica serotype Typhimurium
Source: Sci Rep. 2017 Sep 4;7:10326. doi: 10.1038/s41598-017-10598-2 (PMC5583245; doi:10.1038/s41598-017-10598-2)
Supplement: Supplementary file 1 — Supplementary Figures and Tables [file 41598_2017_10598_MOESM1_ESM.pdf]

Supplementary information for

Functional expression of the entire adhesiome of *Salmonella enterica* serotype Typhimurium

Nicole Hansmeier[1], Katarzyna Miskiewicz[1], Laura Elpers[1], Viktoria Liss[1], Michael Hensel[1,#], Torsten Sterzenbach[1,#]

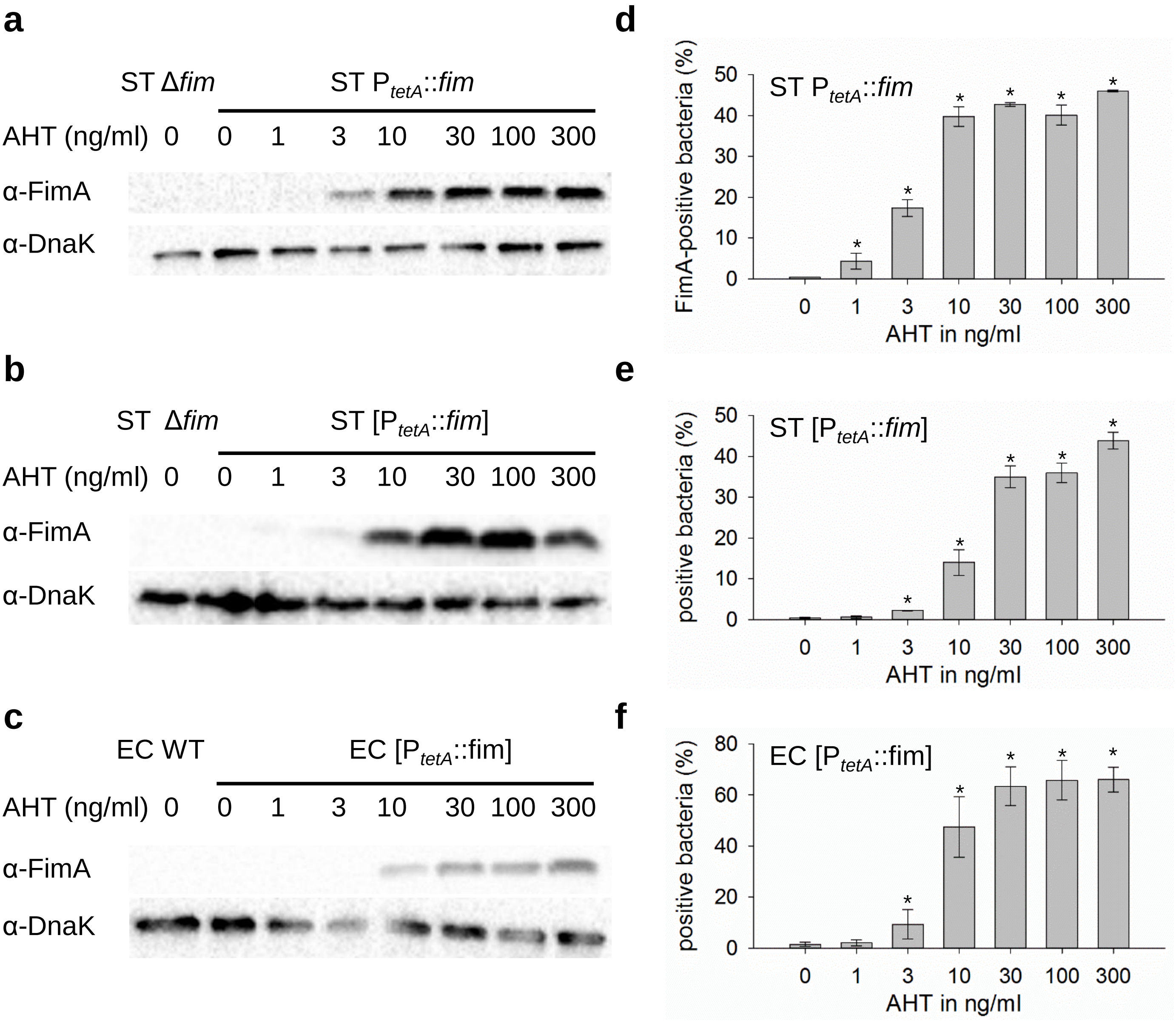

Fig. S1. Controlled expression of type 1 fimbriae. Expression of type 1 fimbriae was induced by addition of various concentrations of AHT in the indicated strains. (a, b and c) Expression of the main subunit of type 1 fimbriae FimA was assessed from bacterial lysates by Western blotting. As loading control, DnaK was detected by Western blot analysis. (d, e and f) Surface expression of type 1 fimbriae in the indicated strains was measured by flow cytometry targeting the main subunit FimA. * $P < 0.05$ (Student's *t*-test, compared to non-induced controls).

Fig. S1

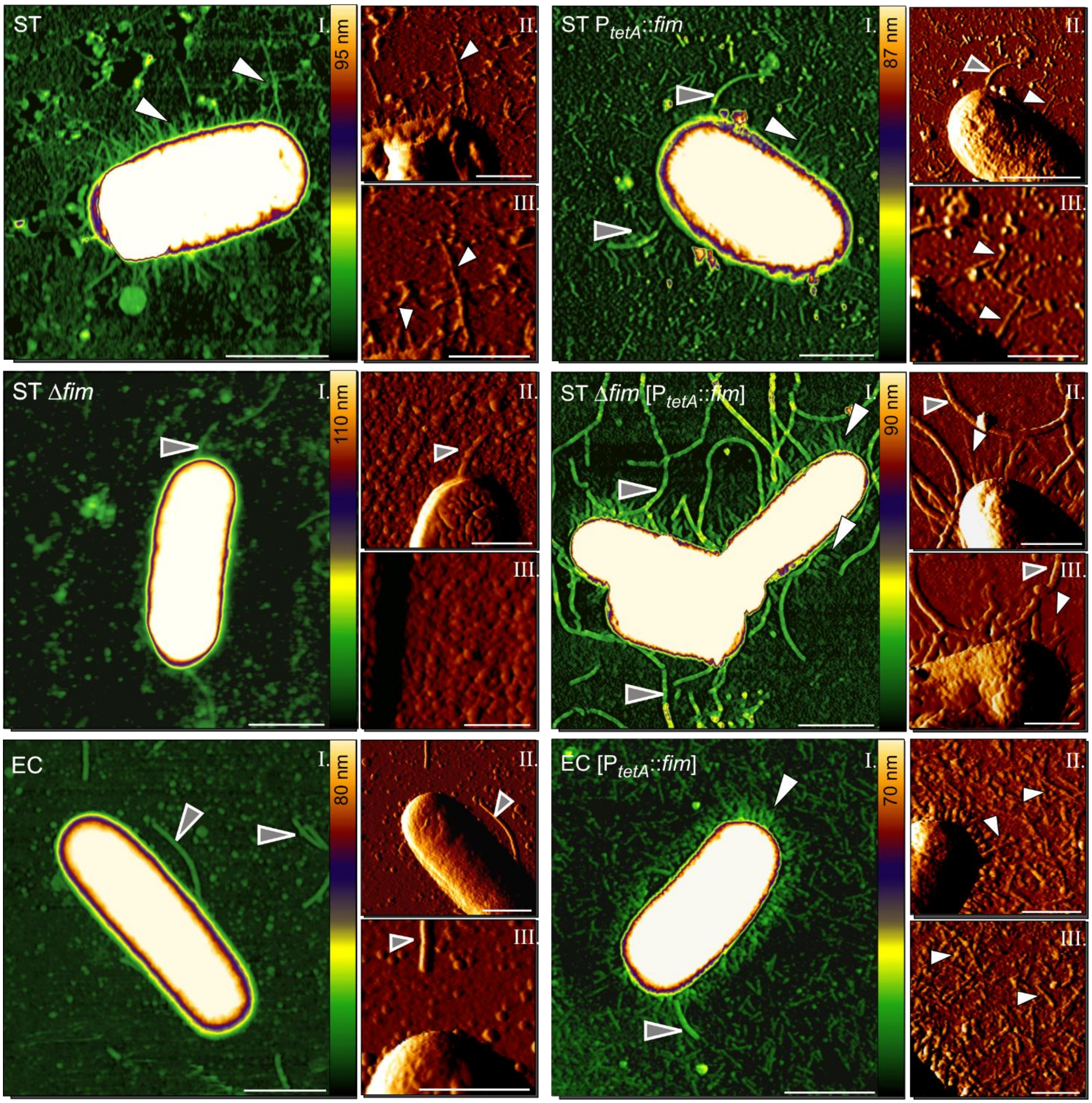

Fig. S2. Visualization of surface expression of type 1 fimbriae. Strains as indicated were grown either statically for 24 h (ST and ST Δ*fim*), or aerobically (all other strains) for 3.5 h in the presence (constructs containing P_{tetA}::*fim*) or absence (other strains) of 100 ng/ml AHT. Expression of type 1 fimbriae was visualized by AFM in the indicated strains. Each panel contains a height image (I) and deflection images (II and III). Color bars indicate the Z-range. White arrow heads point to type 1 fimbriae and grey arrow heads to flagella. Scale bars indicate 1 µm in (I) and 0.5 µm in (II) and (III).

Fig. S2

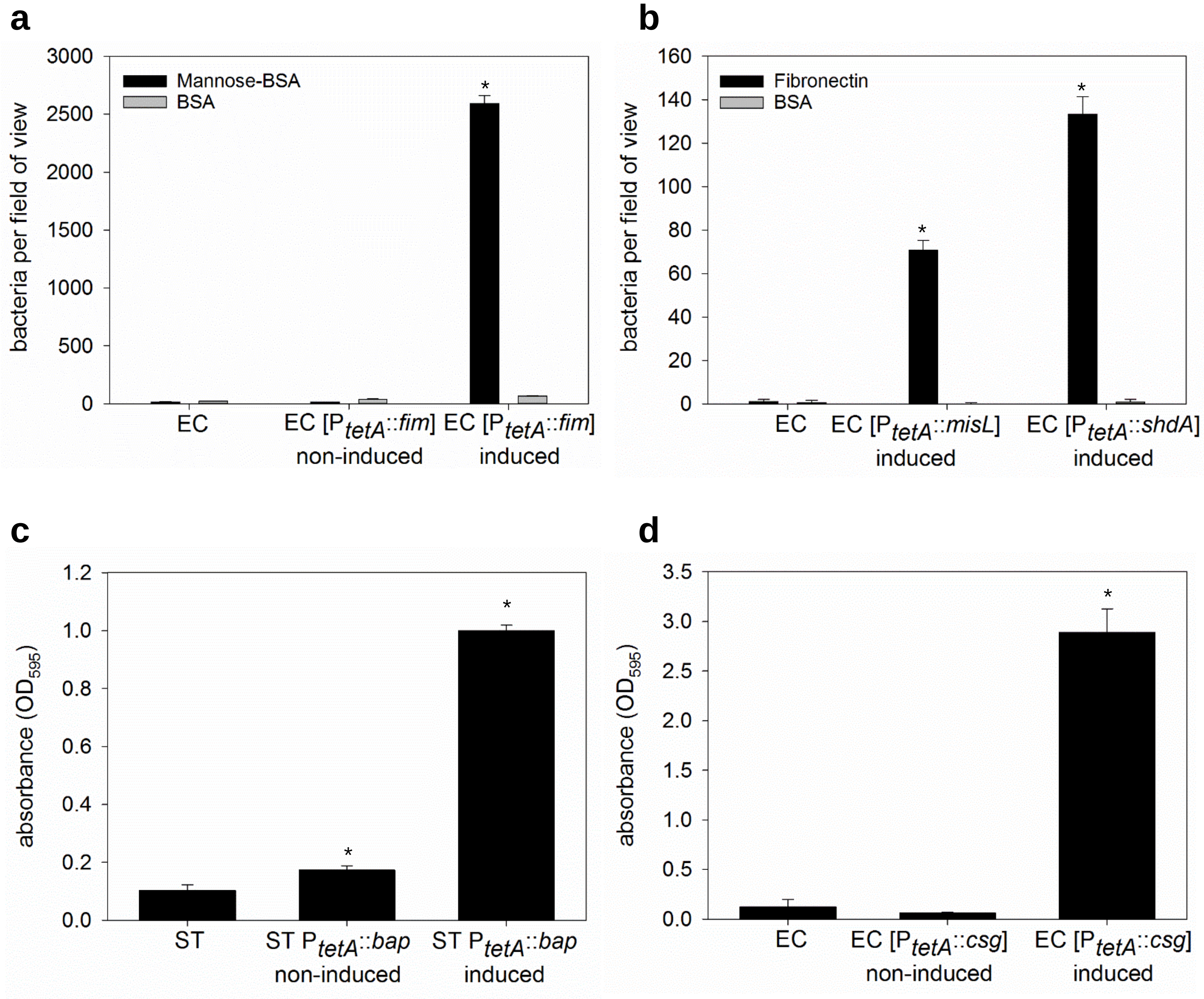

Fig. S3. Functional characterization of heterologously expressed adhesins. (a and b) Plastic surfaces were coated with BSA and either Mannose-BSA (a) or fibronectin (b). WT EC or EC harboring the expression plasmid for type 1 fimbria (a), *misL* or *shdA* (b), with or without induction with 100 ng/ml AHT were added to the coated plates. After removal of non-adherent bacteria, numbers of adherent bacteria were determined using ImageJ. Mean numbers of adherent bacteria per field of view and standard deviations from three independent experiments are shown. (c and d) WT EC or ST or EC or ST harboring the expression plasmids for *bap* (c) or *csg* (d), with or without induction with 100 ng/ml AHT were incubated in 96-well microtiter plates at 30°C for 48 h. After removal of planktonic bacteria, biofilm forming bacteria were stained with 0.1% crystal violet and the absorbance at OD_{595} was measured after solubilization of the dye with 30% acetic acid. * $P < 0.05$ (Student's *t*-test compared to WT bacteria).

Fig. S3

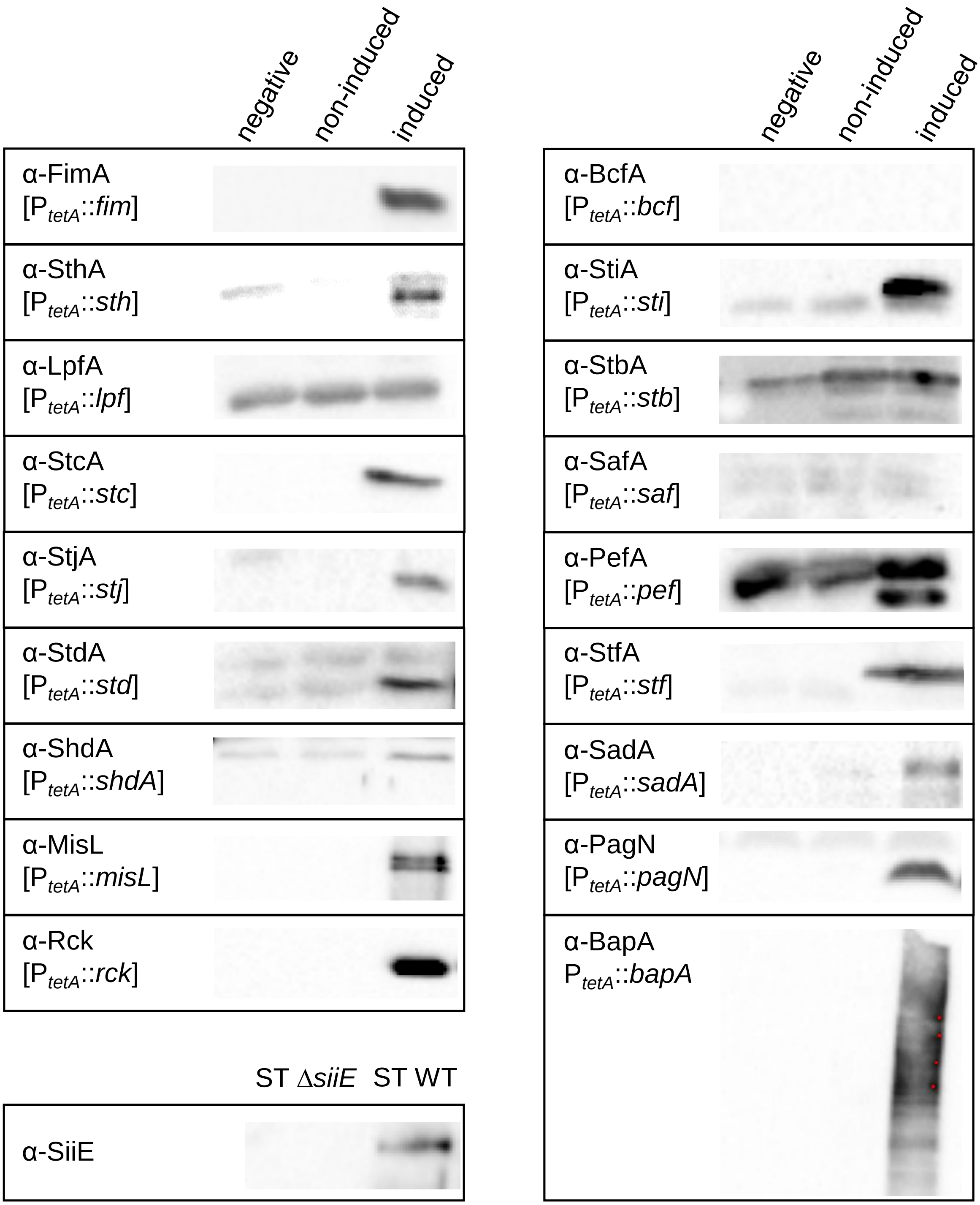

Fig. S4. Expression of adhesins in *E. coli* or *Salmonella*. *E. coli* ORN172 without a plasmid (negative) or harboring the indicated expression plasmid were grown for 3.5 h aerobically in the absence (non-induced) or presence (induced) of 100 ng/ml AHT. For BapA and SiiE, *S.* Typhimurium SR11, a *siiE* mutant or bacteria containing the tetracycline-inducible expression cassette in front of the *bapABCD* operon were grown for 3.5 h in the absence or presence (induced) of 100 ng/ml AHT. Expression of the indicated adhesins was detected by Western blotting from lysates of the indicated strains.

Fig. S4

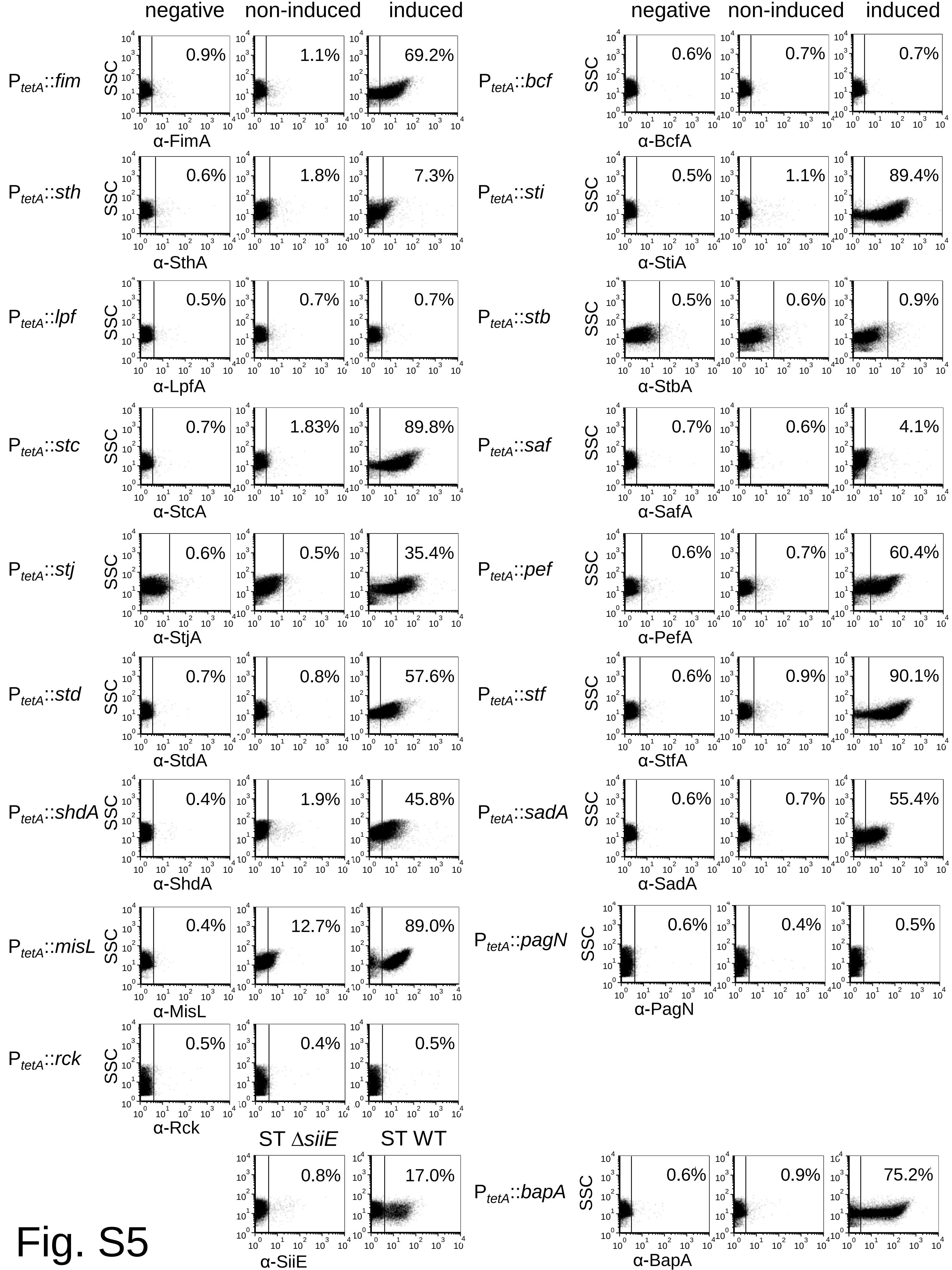

negative
non-induced
induced
PtetA::fim
0.9%
1.1%
69.2%
α-FimA
PtetA::sth
0.6%
1.8%
7.3%
α-SthA
PtetA::lpf
0.5%
0.7%
0.7%
α-LpfA
PtetA::stc
0.7%
1.83%
89.8%
α-StcA
PtetA::stj
0.6%
0.5%
35.4%
α-StjA
PtetA::std
0.7%
0.8%
57.6%
α-StdA
PtetA::shdA
0.4%
1.9%
45.8%
α-ShdA
PtetA::misL
0.4%
12.7%
89.0%
α-MisL
PtetA::rck
0.5%
0.4%
0.5%
α-Rck
ST ΔsiiE
ST WT
0.8%
17.0%
α-SiiE
PtetA::bcf
0.6%
0.7%
0.7%
α-BcfA
PtetA::sti
0.5%
1.1%
89.4%
α-StiA
PtetA::stb
0.5%
0.6%
0.9%
α-StbA
PtetA::saf
0.7%
0.6%
4.1%
α-SafA
PtetA::pef
0.6%
0.7%
60.4%
α-PefA
PtetA::stf
0.6%
0.9%
90.1%
α-StfA
PtetA::sadA
0.6%
0.7%
55.4%
α-SadA
PtetA::pagN
0.6%
0.4%
0.5%
α-PagN
PtetA::bapA
0.6%
0.9%
75.2%
α-BapA
SSC

Fig. S5

Fig. S5. Expression of various *S*. Typhimurium adhesins in *E*. *coli* or *S.* Typhimurium. *E. coli* ORN172 without a plasmid (negative), or containing the indicated expression plasmids were grown for 3.5 h aerobically in the absence (non-induced) or presence (induced) of 100 ng/ml ATH. For expression of BapA, *S.* Typhimurium containing the tetracycline-inducible expression cassette were used. For SiiE, WT *S.* Typhimurium and a *siiE* mutant strain were used. Surface expression of the indicated adhesive structure was assessed by flow cytometry using antisera specific to the expressed adhesin.

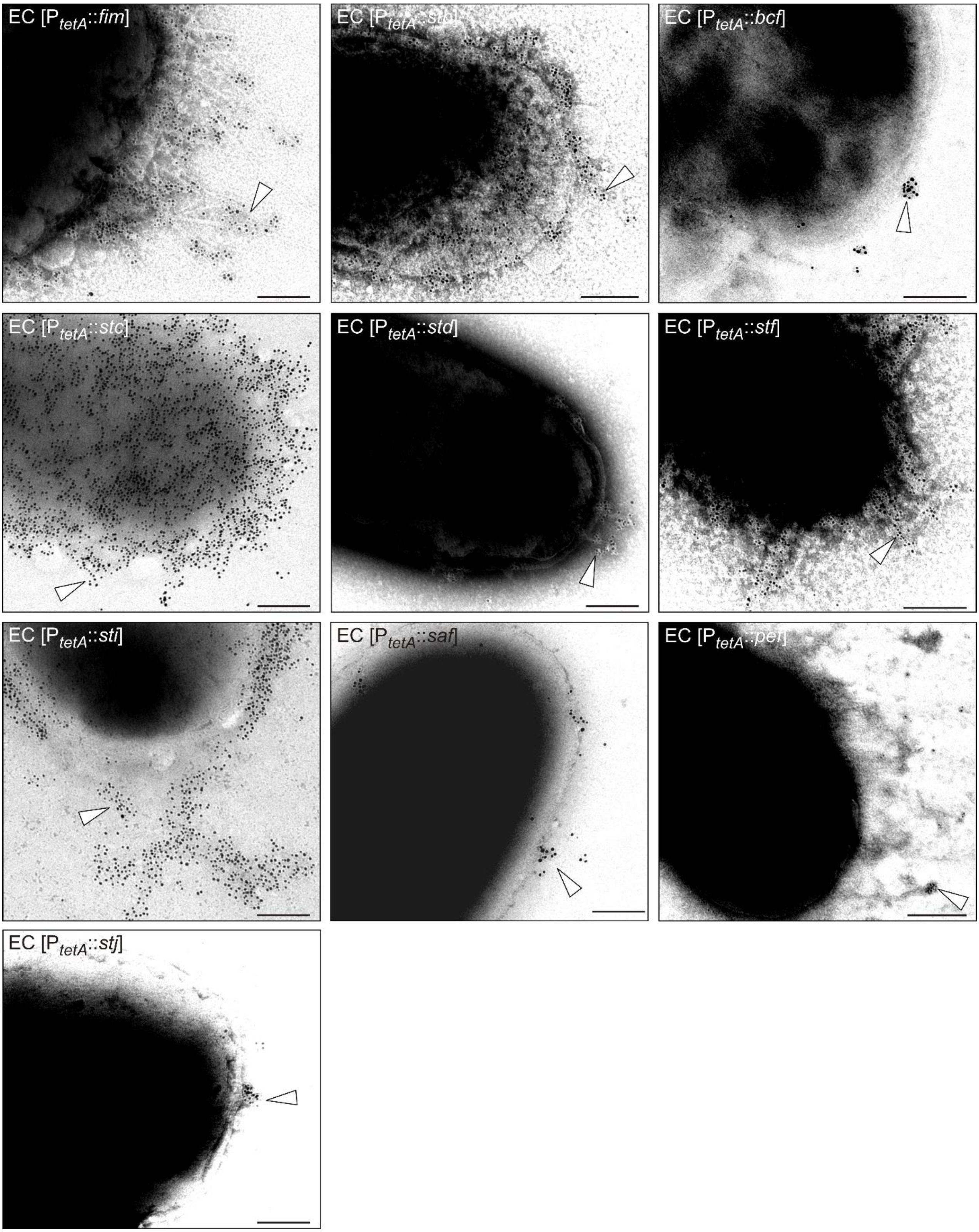

Fig. S6. Immuno-gold labeling with silver enhancement of various fimbrial adhesins of *S*. Typhimurium in *E.coli* ORN172 expressing particular adhesins after induction with 100 ng/ml AHT. Scale bars indicate 0.2 µm.

Fig. S6

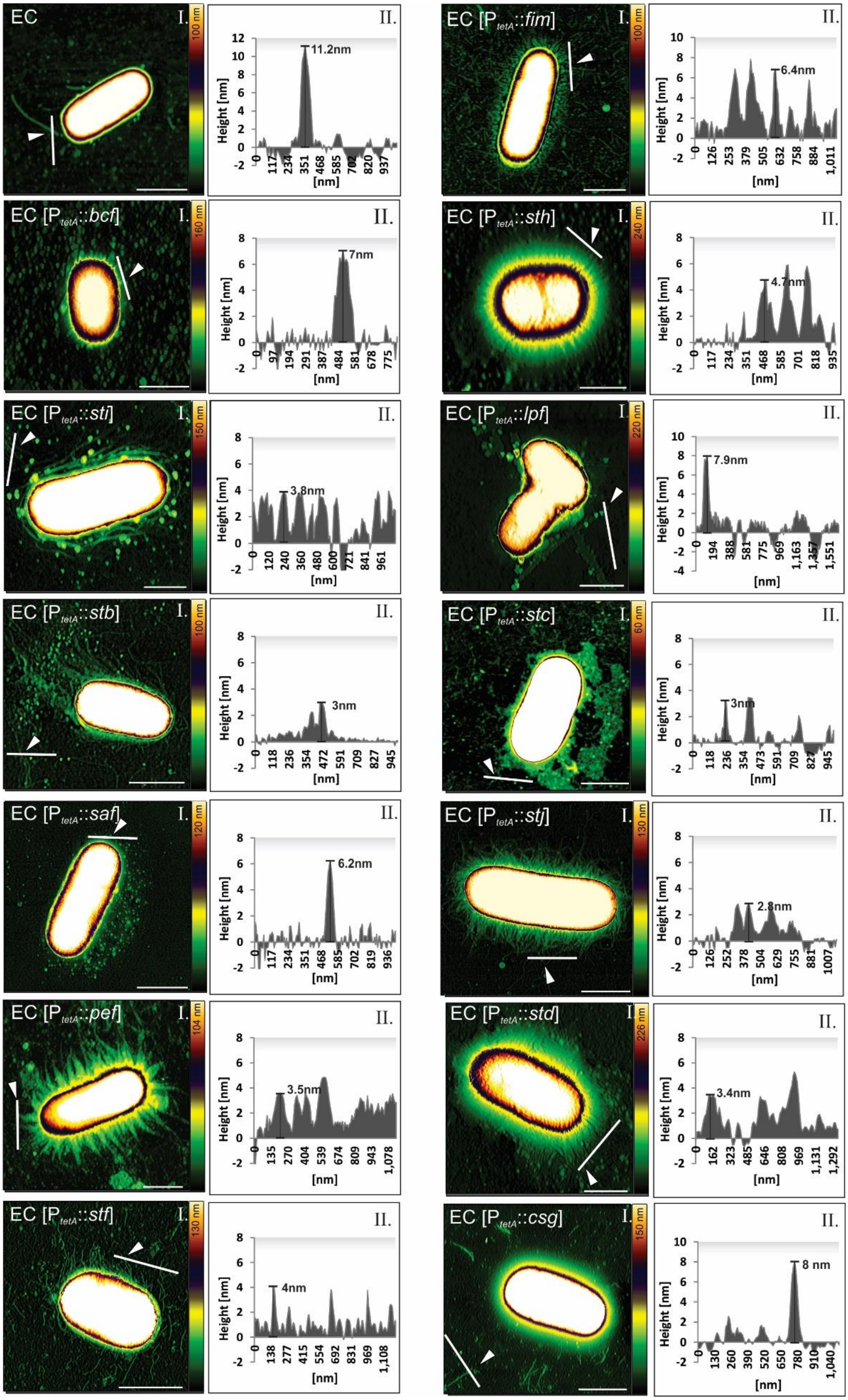
EC
I.
100 nm
II.
11.2nm
Height [nm]
[nm]
EC [P_tetA::fim]
6.4nm
EC [P_tetA::bcf]
160 nm
7nm
EC [P_tetA::sth]
240 nm
4.7nm
EC [P_tetA::sti]
150 nm
3.8nm
EC [P_tetA::lpf]
220 nm
7.9nm
EC [P_tetA::stb]
3nm
EC [P_tetA::stc]
60 nm
3nm
EC [P_tetA::saf]
120 nm
6.2nm
EC [P_tetA::stj]
130 nm
2.8nm
EC [P_tetA::pef]
104 nm
3.5nm
EC [P_tetA::std]
226 nm
3.4nm
EC [P_tetA::stf]
4nm
EC [P_tetA::csg]
8 nm

Fig. S7

Fig. S7. Example height profiles of flagella and fimbrial adhesins. Each panel contains an AFM height image of flagella or fimbrial adhesins expressed in *E. coli* ORN172 (I) and the corresponding height profile (II). Height dimensions of fimbriae were determined after XY tilt correction from raw images and derived from Z-dimensions since X-and Y-measurements are affected by the tip geometry. The positions for the analysis were carefully chosen to ensure that individual fimbria rather than bundles were measured. A white bar in (I) indicates the area of the height profile depicted in (II).

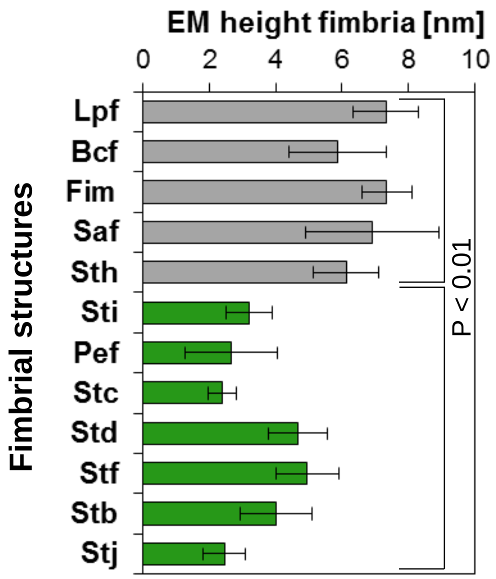

Fig. S8. Characterization of fimbrial adhesins of *S.* Typhimurium. The diameter of 20 singular fimbrial structures were measured from TEM images. The graphs show the average diameter and the standard deviation of the respective fimbrial structure. Grey bars indicate thick (C_{Thick}), and green bars thin (C_{Thin}) fimbriae. Statistical analyses using t-test indicate that these two groups ($C_{Thin;}$ C_{Thick}) are statistically different.

Fig. S8

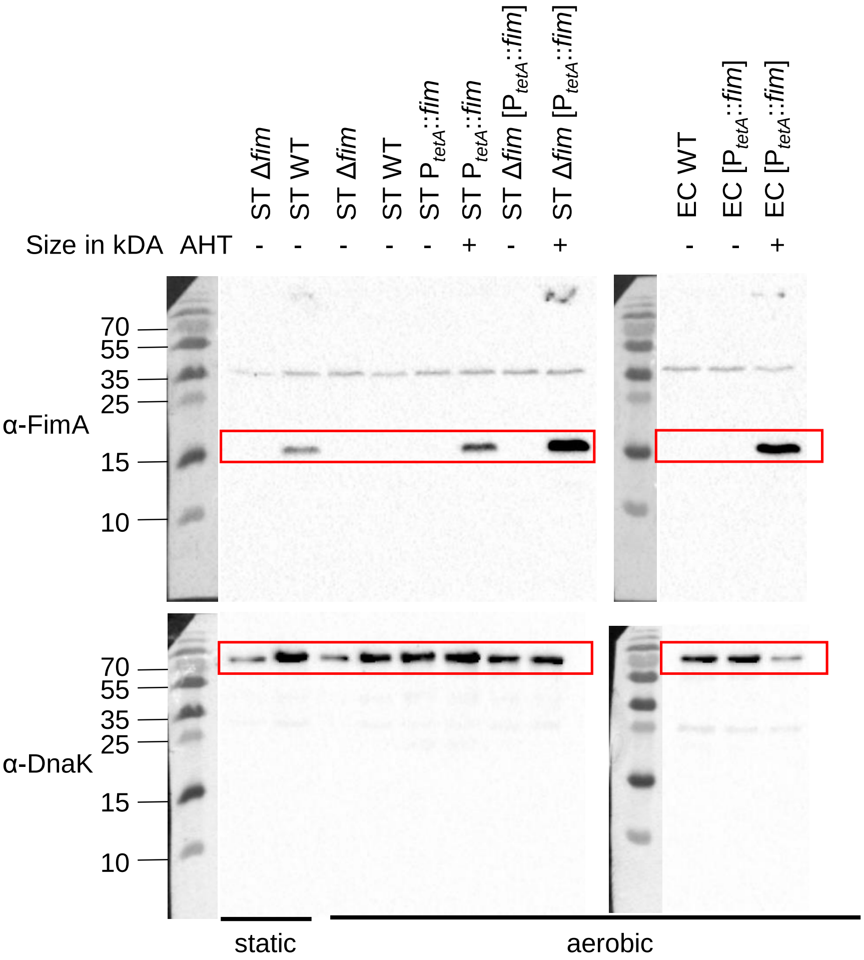

Fig. S9. Uncropped images including molecular size markers of Fig. 1c.

Fig. S9

Table S1. Bacterial strains used in this study.

Designation	Relevant characteristics	Source or reference
E. coli NEB5α	Cloning strain	New England Biolabs
E. coli ORN172	Δ*fimBEACDFGH*::*aph*	[1]
S. Typhimurium NCTC12023	Wild type	NCTC, lab stock
S. Typhimurium LT2	Wild type	[2]
S. Typhimurium SR11	Wild type	[3]
SPN342	SR11 Δ*fimAICDHF*	[4]
MvP599	12023 Δ*siiE*	[5]
MvP2131	12023 *tetR* P_{tetA}::*bapABCD*	This study

Table S2. Plasmids used in this study.

Designation	Relevant genotype	Source or Reference
pWSK29	Low copy number cloning vector	[6]
pWRG99	Vector encoding Redαβγ *tetR* P_{tetA}::I-*Sce*I	[7]
pWRG730	Vector encoding Redαβγ	[8]
p2795	Generic template vector	[9]
p3773	*tetR* P_{tetA} in p2795	This study
p4380	*tetR* P_{tetA}::*csgBACEFG* in pWSK29	This study
p4389	*tetR* P_{tetA}::*stiABCD* in pWSK29	This study
p4390	*tetR* P_{tetA}::*stfABCDEFG* in pWSK29	This study
p4391	*tetR* P_{tetA}::*stbABCDEFG* in pWSK29	This study
p4392	*tetR* P_{tetA}::*fimAICDHF* in pWSK29	This study
p4393	*tetR* P_{tetA}::*safABCD* in pWSK29	This study
p4394	*tetR* P_{tetA}::*stdABCD* in pWSK29	This study
p4395	*tetR* P_{tetA}::*stjABCDE* in pWSK29	This study
p4396	*tetR* P_{tetA}::*pefACDEF* in pWSK29	This study
p4397	*tetR* P_{tetA}::*bcfABCDEFG* in pWSK29	This study
p4519	*tetR* P_{tetA}::*lpfABCDE* in pWSK29	This study
p4399	*tetR* P_{tetA}::*stcABC* in pWSK29	This study
p4400	*tetR* P_{tetA}::*sthABCDE* in pWSK29	This study
p4401	*tetR* P_{tetA}::*pagN* in pWSK29	This study
p4402	*tetR* P_{tetA}::*rck* in pWSK29	This study
p4403	*tetR* P_{tetA}::*misL* in pWSK29	This study
p4404	*tetR* P_{tetA}::*sadA* in pWSK29	This study
p4520	*tetR* P_{tetA}::*shdA* in pWSK29	This study

Table S3**.** Antibodies used in this study.

Target	Antibody raised against	Reference/Source
α-BcfA	recombinant GST-BcfA	[10]
α-FimA	recombinant GST-FimA	[10]
α-LpfA	recombinant GST-LpfA	[10]
α-PefA	recombinant GST-PefA	[10]
α-SafA	recombinant GST-SafA	[10]
α-StbA	recombinant GST-StbA	[10]
α-StcA	recombinant GST-StcA	[10]
α-StdA	recombinant GST-StdA	[10]
α-StfA	recombinant GST-StfA	[10]
α-SthA	recombinant GST-SthA	[10]
α-StiA	recombinant GST-StiA	[10]
α-StjA	recombinant GST-StjA	[10]
α-MisL	recombinant GST-$MisL_{29\text{-}875}$	[11]
α-SadA	recombinant SadA	[12]
α-ShdA	recombinant GST-ShdA	[13]
α-BapA	N-terminal BapA fragment	[14]
α-SiiE	C-terminal SiiE fragment	[5]
α-PagN	recombinant MBP-PagN	[15]
α-Rck	recombinant Rck	[16]
α-DnaK	*E. coli* DnaK	Enzo Lifesciences

Table S4. Oligonucleotides used in this study.

Name	Sequence	Purpose
TetR-PtetA-For-SacI	GCGGAGCTCCACTCGAACTGCATACAGTAGG	
TetR-PtetA-Rev-XhoI	TATCTCGAGGGAAAAAGGTTATGCTGCTTTTA	
tetR P_{tetA} fusion to adhesins		
Ptet-stb-fw	agttaatgatcgttatttttaccactcctccataagcacgGTACCGTGTAGGCTGGAGC	*stb*
Ptet-stb-rv	cctgtattaatctttactttttcaggacgagtgcaattcgCATTACCTGGTTTTTTTGATGC	
Ptet-sth-fw	aagcgcatacagagtaaaatataatattttattttatatgGTACCGTGTAGGCTGGAGC	*sth*
Ptet-sth-rv	aattgttgcctgattctattcgctgaaaaataaagatagcCATTACCTGGTTTTTTTGATGC	
Ptet-stf-fw	tctactaatataaacatggggtattgagtataactctgtgGTACCGTGTAGGCTGGAGC	*stf*
Ptet-stf-rv	tcatccttattaaaagttggtgagtatttttacgctattcCATTACCTGGTTTTTTTGATGC	
Ptet-sti-fw	atttactcattcgggaataaaaagaacaataactttccacGTACCGTGTAGGCTGGAGC	*sti*
Ptet-sti-rv	taagatattataaatattgacatagtaacaatatctatagCATTACCTGGTTTTTTTGATGC	
Ptet-bcf-fw	agtcgtgatattgctgtgaagaaatatcagcagccgtttcGTACCGTGTAGGCTGGAGC	*bcf*
Ptet-bcf-rv	ccttttaaatataaaaataagggtaatcagatttttttaacCATTACCTGGTTTTTTTGATGC	
Ptet-saf-fw	gtacaagctgttattaccagccacggatttttttacatacgGTACCGTGTAGGCTGGAGC	*saf*
Ptet-saf-rv	ccagcacatccagaatacataacgccataccaaatcttacCATTACCTGGTTTTTTTGATGC	
Ptet-stc-fw	gtgtttacattgcgataacttcctgtctatgagaattttcGTACCGTGTAGGCTGGAGC	*stc*
Ptet-stc-rv	aagtaatttctatttgttaagagttattaacccttgcaacCATTACCTGGTTTTTTTGATGC	
Ptet-stj-fw	tttaattttattaaatttacaacatatcattatcttcataGTACCGTGTAGGCTGGAGC	*stj*

Ptet-stj-rv	gattaagtatatttccaaatgacatgtaatgcgcgggtcgCATTACCTGGTTTTTTTGATGC	
Ptet-lpf-fw	tatactaattatagtatccaatacccacctctatacactcGTACCGTGTAGGCTGGAGC	*lpf*
Ptet-lpf-rv	gaacgcatccttaggattatctgcattctgtgaggaaatgCATTACCTGGTTTTTTTGATGC	
Ptet-PfimA-fw	gaaatgtttaatttattaccgtgacgaaatgtcatattcgGTACCGTGTAGGCTGGAGC	*fim*
Ptet-PfimA-rv	gtttcatggatttcccttgaattacacacacccggtttcgCATTACCTGGTTTTTTTGATGC	
Ptet-Pstd-fw	acaccaggcgtttattattcatacgaatcttttctgaacgGTACCGTGTAGGCTGGAGC	*std*
Ptet-Pstd-rv	aatatgtcctttgggtgaatgagaattattttgcaaaggcCATTACCTGGTTTTTTTGATGC	
Ptet-Ppef-fw	cggatggtaactcaggatttttacgatgtcacgtcatagcGTACCGTGTAGGCTGGAGC	*pef*
Ptet-Ppef-rv	caaaatgaaaatacacattcacattttccagcatggctggCATTACCTGGTTTTTTTGATGC	
Ptet-PbapA-fw	acgggaaggctcgtctacgcattttgccctgaacgttgtgcGTACCGTGTAGGCTGGAGC	*bap*
Ptet-PbapA_rv	cataaatcagctcctgatggatttgctctgtgtattaattaacgCATTACCTGGTTTTTTTGATGC	
**Primer for Gibson assembly**		
Inverse PCR from pWSK29 for GA		
Vf-pWSK29	GAATTCCTGCAGCCCGGGG	
Vr-pWSK29	AAGCTTATCGATACCGTCGACCTC	
Forward primer for amplification of fimbrial operons including Tet cassette		
1f-ST_Ptet-pWSK29	TCGACGGTATCGATAAGCTTAGGGAAAAAGGTTATGCTGCT	
Reverse primers for amplification of various operons		
1r-ST_Ptet-fim-pWSK29	CCCGGGCTGCAGGAATTCTACTCCCGGCGAATTATCGT	*fim*
1r-ST_Ptet-stb-pWSK29	CCGGGCTGCAGGAATTCGCTTGCCTCAGGGATACG	*stb*

1r-ST_Ptet-sth-pWSK29	CCGGGCTGCAGGAATTCagcgccagaaactgtgatgc	*sth*
1r-ST_Ptet-stf-pWSK29	CCGGGCTGCAGGAATTCCGTCCAGAAACAACGTAG	*stf*
1r-ST_Ptet-sti-pWSK29	CCGGGCTGCAGGAATTCGACTGGGGAGATGGGGCGTTG	*sti*
1r-ST_Ptet-bcf-pWSK29	CCGGGCTGCAGGAATTCGGTGAGAACATTTTTCATAATATT	*bcf*
1r-ST_Ptet-saf-pWSK29	CCGGGCTGCAGGAATTCCGAACCTGATATACAGTATTC	*saf*
1r-ST_Ptet-stc-pWSK29	CCGGGCTGCAGGAATTCCCTTTTGAAACTACCGCATAC	*stc*
1r-ST_Ptet-stj-pWSK29	CCGGGCTGCAGGAATTCGTGAAGGGGGCCAGAGCAGG	*stj*
1r-ST_Ptet-lpf-pWSK29	CCGGGCTGCAGGAATTCGGCAACGGAGAGTGTGAT	*lpf*
1r-ST_Ptet-std-pWSK29	CCGGGCTGCAGGAATTCGTGTGTTTATTCGGGATTAG	*std*
1r-ST_Ptet-pef-pWSK29	CCGGGCTGCAGGAATTCCCTCAGTACACCACGCATGG	*pef*
Amplification of diverse genes or operons		
Vf-pWSK29-Ptet	GAATTCCTGCAGCCCGGGG	Inverse PCR from p4392 for GA
Vr-pWSK29-Ptet	CATTACCTGGTTTTTTTGATGCATTTCACT	
1f-ST-Ptet-rck-pWSK29	TGCATCAAAAAAACCAGGTAATGGAACTTAACTGTGTTCAGGGAGTTTTA	Amplification of *rck* for GA
1r- ST-Ptet-rck-pWSK29	CCCGGGCTGCAGGAATTCTGCGGCTCCGCTCCCTTT	
1f-ST-Ptet-pagN-pWSK29	TGCATCAAAAAAACCAGGTAATGCAATATTAAGGCAGGTTCTG	Amplification of *pagN* for GA
1r-ST-Ptet-pagN-pWSK29	CCGGGCTGCAGGAATTCTTAAAAGGCGTAAGTAATGC	
1f-ST-Ptet-misL-pWSK29	TGCATCAAAAAAACCAGGTAATGCGCCATAATGCAGGAGGC	Amplification of *misL* for GA
1r-ST-Ptet-misL-pWSK29	CCGGGCTGCAGGAATTCAGCGGCTCTGTTGTTACC	
1f-ST-Ptet-shdA-pWSK29	TGCATCAAAAAAACCAGGTAATGTTACAGTATTGTCTGGAGCGCCGTGC	Amplification of *shdA* for GA

1r-ST-Ptet-shdA-pWSK29	CCGGGCTGCAGGAATTCATCTGACGATCAACCGGTTTGTC	
1f-ST-Ptet-sadA-pWSK29	TGCATCAAAAAAACCAGGTAATGTACAATTATTTTAGAAAAGGAAATTACT	Amplification of *sadA* for GA
1r-ST-Ptet-sadA-pWSK29	CCGGGCTGCAGGAATTCATGGCATTATGCCATTGC	
1f-Ptet-STM1143-45-pWSK29	TGCATCAAAAAAACCAGGTAATGTACGACCAGGTCCAGGGT	Amplification of *csgBAC* for GA
1r-Ptet-STM1143-45-pWSK29	CCTTACCGCCCATCAAAAACTACTGTGCAGAAGG	
2f-Ptet-STM1141-39-pWSK29	GTTTTTGATGGGCGGTAAGGCCATGAAACGC	Amplification of *csgEFG* for GA
2r-Ptet-STM1141-39-pWSK29	CCGGGCTGCAGGAATTCCGTGGGGTTCTTCCCCACGCT	

Supplementary References

1. Woodall, L.D., Russell, P.W., Harris, S.L. & Orndorff, P.E. Rapid, synchronous, and stable induction of type 1 piliation in *Escherichia coli* by using a chromosomal lacUV5 promoter. *J Bacteriol* **175**, 2770-8 (1993).
2. McClelland, M. *et al.* Complete genome sequence of *Salmonella enterica* serovar Typhimurium LT2. *Nature* **413**, 852-6 (2001).
3. Schneider, H.A. & Zinder, N.D. Nutrition of the host and natural resistance to infection. V. An improved assay employing genetic markers in the double strain inoculation test. *J Exp Med* **103**, 207-23 (1956).
4. Sterzenbach, T. *et al.* A novel CsrA titration mechanism regulates fimbrial gene expression in *Salmonella typhimurium*. *EMBO J* **32**, 2872-83 (2013).
5. Gerlach, R.G. *et al. Salmonella* Pathogenicity Island 4 encodes a giant non-fimbrial adhesin and the cognate type 1 secretion system. *Cell Microbiol* **9**, 1834-50 (2007).
6. Wang, R.F. & Kushner, S.R. Construction of versatile low-copy-number vectors for cloning, sequencing and gene expression in *Escherichia coli*. *Gene* **100**, 195-9 (1991).
7. Blank, K., Hensel, M. & Gerlach, R.G. Rapid and highly efficient method for scarless mutagenesis within the *Salmonella enterica* chromosome. *PLoS One* **6**, e15763 (2011).
8. Hoffmann, S., Schmidt, C., Walter, S., Bender, J.K. & Gerlach, R.G. Scarless deletion of up to seven methyl-accepting chemotaxis genes with an optimized method highlights key function of CheM in *Salmonella* Typhimurium. *PLoS One* **12**, e0172630 (2017).
9. Husseiny, M.I. & Hensel, M. Rapid method for the construction of *Salmonella enterica* Serovar Typhimurium vaccine carrier strains. *Infect Immun* **73**, 1598-605 (2005).
10. Humphries, A.D. *et al.* The use of flow cytometry to detect expression of subunits encoded by 11 *Salmonella enterica* serotype Typhimurium fimbrial operons. *Mol Microbiol* **48**, 1357-76 (2003).
11. Dorsey, C.W., Laarakker, M.C., Humphries, A.D., Weening, E.H. & Baumler, A.J. *Salmonella enterica* serotype Typhimurium MisL is an intestinal colonization factor that binds fibronectin. *Mol Microbiol* **57**, 196-211 (2005).
12. Hartmann, M.D. *et al.* Complete fiber structures of complex trimeric autotransporter adhesins conserved in enterobacteria. *Proc Natl Acad Sci U S A* **109**, 20907-12 (2012).
13. Kingsley, R.A., Santos, R.L., Keestra, A.M., Adams, L.G. & Baumler, A.J. *Salmonella enterica* serotype Typhimurium ShdA is an outer membrane fibronectin-binding protein that is expressed in the intestine. *Mol Microbiol* **43**, 895-905 (2002).
14. Latasa, C. *et al.* BapA, a large secreted protein required for biofilm formation and host colonization of *Salmonella enterica* serovar Enteritidis. *Mol Microbiol* **58**, 1322-39 (2005).
15. Lambert, M.A. & Smith, S.G. The PagN protein of *Salmonella enterica* serovar Typhimurium is an adhesin and invasin. *BMC Microbiol* **8**, 1-11 (2008).
16. Rosselin, M. *et al.* Rck of *Salmonella enterica*, subspecies enterica serovar enteritidis, mediates zipper-like internalization. *Cell Res* **20**, 647-64 (2010).
